# CDCA7 and HELLS suppress DNA:RNA hybrid-associated DNA damage at pericentromeric repeats

**DOI:** 10.1038/s41598-020-74636-2

**Published:** 2020-10-20

**Authors:** Motoko Unoki, Jafar Sharif, Yuichiro Saito, Guillaume Velasco, Claire Francastel, Haruhiko Koseki, Hiroyuki Sasaki

**Affiliations:** 1 Division of Epigenomics and Development, Medical Institute of Bioregulation, Kyushu University, 3-1-1 Maidashi, Higashi-ku, Fukuoka-shi, Fukuoka, 812-8582 Japan; 2 Laboratory for Developmental Genetics, RIKEN Center for Integrative Medical Sciences, Kanagawa, 230-0045 Japan; 3 Department of Chromosome Science, National Institute of Genetics, Research Organization of Information and Systems (ROIS), Mishima, Shizuoka, 411-8540 Japan; 4 CNRS UMR7216, Epigenetics and Cell Fate, Université Paris Diderot, Sorbonne Paris Cité, 75205 Paris, France

**Keywords:** Diseases, Chromosomes, DNA damage and repair, Epigenetics

## Abstract

Immunodeficiency, centromeric instability, facial anomalies (ICF) syndrome is a rare autosomal recessive disorder that is caused by mutations in either *DNMT3B*, *ZBTB24*, *CDCA7*, *HELLS*, *or* yet unidentified gene(s). Previously, we reported that the CDCA7/HELLS chromatin remodeling complex facilitates non-homologous end-joining. Here, we show that the same complex is required for the accumulation of proteins on nascent DNA, including the DNMT1/UHRF1 maintenance DNA methylation complex as well as proteins involved in the resolution or prevention of R-loops composed of DNA:RNA hybrids and ssDNA. Consistent with the hypomethylation state of pericentromeric repeats, the transcription and formation of aberrant DNA:RNA hybrids at the repeats were increased in ICF mutant cells. Furthermore, the ectopic expression of RNASEH1 reduced the accumulation of DNA damage at a broad range of genomic regions including pericentromeric repeats in these cells. Hence, we propose that hypomethylation due to inefficient DNMT1/UHRF1 recruitment at pericentromeric repeats by defects in the CDCA7/HELLS complex could induce pericentromeric instability, which may explain a part of the molecular pathogenesis of ICF syndrome.

## Introduction

Immunodeficiency, centromeric instability, and facial anomalies (ICF) syndrome is a rare autosomal recessive disorder characterized by reduced immunoglobulin levels in the serum and recurrent infections^1^. Centromeric instability manifests as stretched heterochromatin, chromosome breaks, and multiradial configurations involving the pericentromeric regions of chromosomes 1, 9, and 16 of activated lymphocytes^2^. These cytological defects are accompanied by DNA hypomethylation in pericentromeric satellite-2 and satellite-3 repeats in these chromosomes.

ICF syndrome patients are categorized into five subtypes: ICF1, ICF2, ICF3, ICF4, and ICFX. Approximately half of ICF patients possess mutations in the *DNA methyltransferase 3B* (*DNMT3B*) gene and are categorized as ICF syndrome type 1 (ICF1, OMIM#242860)^3–5^. In ICF1 cells, DNA hypomethylation is observed in pericentromeric repeats and subtelomeres, the latter of which are vulnerable to DNA damage due to elevated DNA:RNA hybrids^6,7^. In contrast, the other ICF patients—type 2 (ICF2, OMIM#614069), 3 (ICF3, OMIM#616910), 4 (ICF4, OMIM#616911), and X (ICFX)—display hypomethylation in centromeric α-satellite repeats besides hypomethylation in pericentromeric repeats^7^, but not in subtelomeres^8^. ICF2, ICF3, and ICF4 patients possess mutations in the *zinc finger and BTB domain containing 24* (*ZBTB24*), *cell division cycle associated 7* (*CDCA7*), and *helicase lymphoid specific* (*HELLS*) genes, respectively^9,10^. However, the causative gene for the ICFX subtype is still unknown. Among the ICF2–ICF4 genes, *ZBTB24* encodes a protein that transcriptionally activates *CDCA7*^11^. Proteins encoded by *CDCA7* (also known as *JPO1*) and *HELLS* (also known as *LSH*, *PASG*, or *SMARCA6*) constitute a chromatin remodeling complex^12^ and assist in non-homologous end-joining (NHEJ)^13^ and also in homologous recombination (HR) of heterochromatin^14^.

Besides centromeric repeats, pericentromeric repeats, and subtelomeres, a recent study determined that regions with heterochromatic and late replicating signatures are hypomethylated in ICF2, ICF3, and ICF4 patients^15^. It has also been revealed that auxiliary proteins involved in maintenance DNA methylation in early and late replicating regions are partially different^16^. According to the report, the maintenance DNA methylation complex, which includes DNA (cytosine-5)-methyltransferase 1 (DNMT1) and Ubiquitin-like containing PHD and RING finger domains 1 (UHRF1), mediates full methylation at hemimethylated sites in early replicating regions through the dual monoubiquitination of PCNA-associated factor of 15 kDa (PAF15); the same complex targets hemimethylated DNA in late replicating regions through the dual monoubiquitination of the histone H3 tail. Considering that DNMT1 does not prefer hemimethylated DNA wrapped around nucleosomes^17^, we hypothesized that the CDCA7/HELLS complex could facilitate access of the DNMT1/UHRF1 complex to late replicating regions such as pericentromeric repeats to mediate maintenance DNA methylation via chromatin remodeling^12^.

To confirm our hypothesis, we conducted the isolation of proteins on nascent DNA (iPOND)–tandem mass spectrometry (MS/MS) analysis^18^. Through these analyses, we discovered that the accumulation of DNMT1 and UHRF1, as well as proteins involved in the resolution or prevention of R-loops composed of DNA:RNA hybrids and the associated single-strand DNAs, were decreased on nascent DNA in *CDCA7* KO cells. We demonstrated that abnormal transcription from hypomethylated pericentromeric satellite repeats and the formation of aberrant DNA:RNA hybrids occur in ICF mutant cells and presumably trigger DNA damage. Our findings suggest that the CDCA7/HELLS complex mediates a multi-layered protection mechanism by regulating maintenance DNA methylation, the resolution or prevention of DNA:RNA hybrids (R-loops), and DNA repair at pericentromeric satellite repeats. Therefore, the disruption of this mechanism in ICF mutant cells could plausibly contribute to the molecular pathogenesis of ICF syndrome.

## Results

### Proteins involved in maintenance DNA methylation and R-loop resolution/prevention are decreased on nascent DNA in the absence of CDCA7

**Figure 1:**
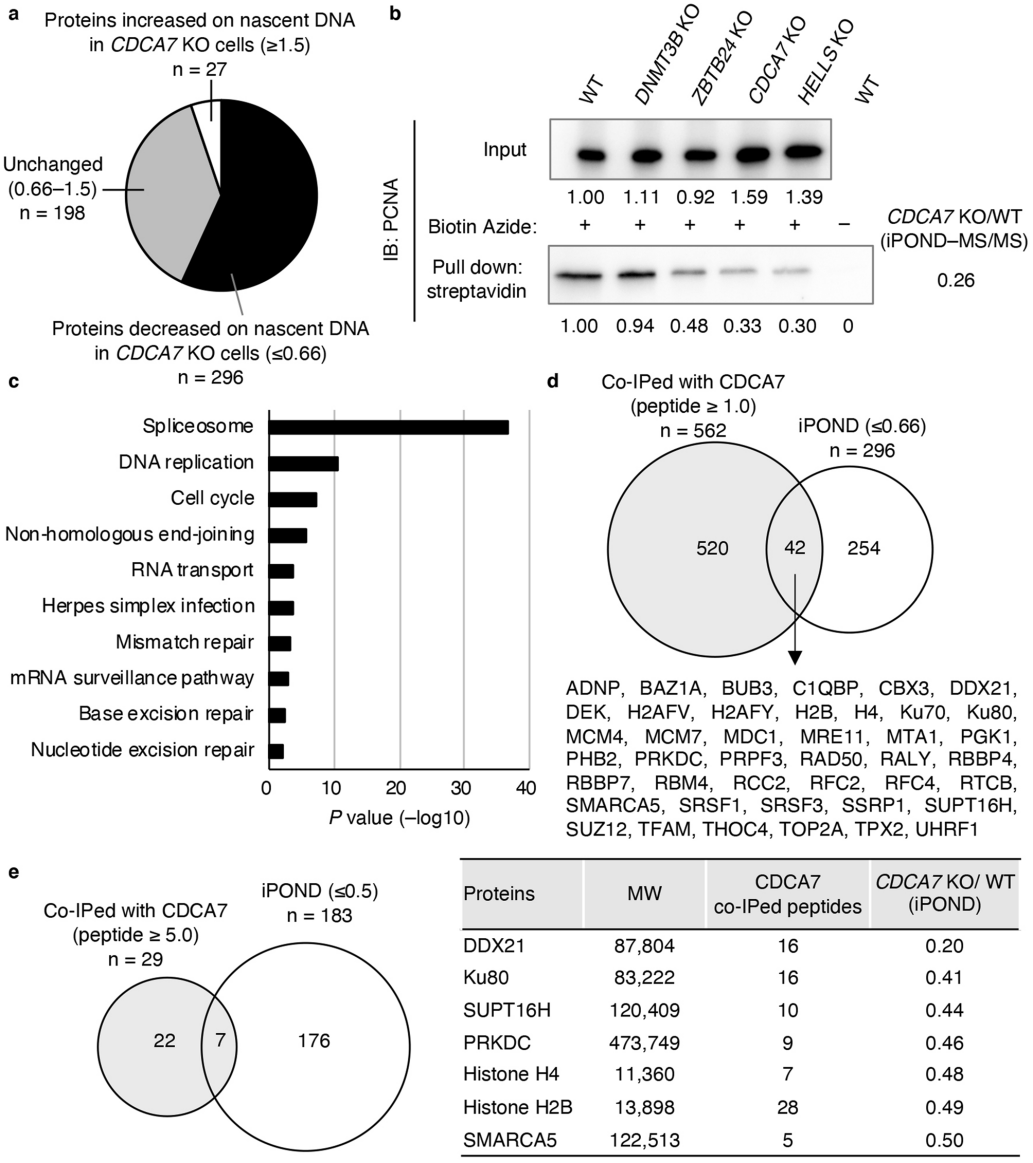
Proteins involved in maintenance DNA methylation and R-loop resolution/prevention are decreased on nascent DNA in the absence of CDCA7. (**a**) Proteins decreased (≤ 0.66, n = 296, Supplementary Table S1), increased (≥ 1.5, n = 27, Supplementary Table S2), or unchanged (0.66–1.5, n = 198) on nascent DNA in *CDCA7* knockout (KO) HEK293 cells compared with wild-type (WT) HEK293 cells, as determined by the isolation of proteins on nascent DNA (iPOND)–tandem mass spectrometry (MS/MS) analysis. (**b**) Confirmation of iPOND–MS/MS analysis by iPOND–Western blotting using an anti-PCNA antibody. (**c**) KEGG pathway analysis of 296 proteins, which were decreased on nascent DNA in *CDCA7* KO cells (Supplementary Table S3). (**d**) Comparison of proteins identified by iPOND–MS/MS analysis (≤ 0.66, n = 296) and proteins that co-immunoprecipitated (co-IPed) with CDCA7 WT protein (peptide ≥ 1.0, n = 562)^13^. Forty-two proteins co-IPed with CDCA7 were decreased on nascent DNA in *CDCA7* KO cells (Supplementary Table S4). (**e**) Comparison of proteins identified by iPOND–MS/MS analysis (≤ 0.5, n = 183) and proteins that co-IPed with CDCA7 WT protein (peptide ≥ 5.0, n = 29)^13^. Seven proteins co-IPed with CDCA7 were decreased on nascent DNA in *CDCA7* KO cells (Supplementary Table S4).

We hypothesized that the CDCA7/HELLS complex could play a role in facilitating maintenance DNA methylation at pericentromeric repeats by recruiting DNA methylation maintenance factors. To confirm this hypothesis, we conducted iPOND–MS/MS analysis using wild-type (WT) and *CDCA7* KO human embryonic kidney (HEK) 293 cells that were previously generated with CRISPR/Cas9-mediated gene editing^13^. iPOND is essentially a reverse chromatin immunoprecipitation. Briefly, 5-ethynyl-2′-deoxyuridine (EdU), which contains an alkyne, was incorporated into newly synthesized nascent DNA in place of thymidine, proteins and DNA were cross-linked using formaldehyde, and biotin was conjugated to the incorporated EdU via the azide-alkyne cycloaddition. Then, proteins on nascent DNA were pulled down by streptavidin agarose beads and subjected to MS/MS analysis. We detected 521 nascent DNA-associated proteins from the analysis. Among these, 296 proteins exhibited decreased accumulation on nascent DNA in *CDCA7* KO cells (≤ 0.66-fold compared with the WT); we confirmed that the expression of several key proteins among the 296 proteins, including UHRF1, DExD-Box Helicase 21 (DDX21), and SPT16 homolog facilitates chromatin remodeling subunit (SUPT16H), was almost the same in the input of WT and *CDCA7* KO cells, which excludes the possibility that these proteins were only decreased in the KO cells (Supplementary Fig. S1). The levels of 198 proteins were unchanged (0.66-fold to 1.5-fold), whereas the levels of 27 proteins were increased (≥ 1.5-fold) (Fig. 1a and Supplementary Tables S1 and S2). Notably, the levels of proliferating cell nuclear antigen (PCNA), which is a key regulator of DNMT1 and UHRF1 at the replication fork, were also decreased in *CDCA7* KO cells (*CDCA7* KO/WT = 0.26). We confirmed the decrease of PCNA on nascent DNA by iPOND combined with Western blotting in place of MS/MS analysis (Fig. 1b). Interestingly, the PCNA accumulation was also decreased in *ZBTB24* KO and *HELLS* KO cells, but not in *DNMT3B* KO cells (Fig. 1b). A Kyoto Encyclopedia of Genes and Genomes (KEGG) pathway analysis revealed that the accumulation of proteins involved in the spliceosome (*P* = 2.0 × 10^−37^), DNA replication (*P* = 3.1 × 10^−11^), cell cycle (*P* = 6.1 × 10^−8^), and NHEJ (*P* = 2.2 × 10^−6^) was significantly decreased in the *CDCA7* KO cells (Fig. 1c and Supplementary Table S3). Importantly, DNMT1 and UHRF1 were included in the 296 proteins exhibited decreased accumulation on nascent DNA (*CDCA7* KO/WT = 0.52 and 0.60, respectively, Supplementary Table S1). This result is consistent with our hypothesis that CDCA7 could be involved in maintenance DNA methylation in specific genomic regions, possibly heterochromatic and late replicating regions^15,16^.

To determine the proteins that are direct targets of CDCA7 on nascent DNA, we compared the 296 proteins that exhibited decreased accumulation on nascent DNA (≤ 0.66, iPOND–MS/MS) to 562 proteins that co-immunoprecipitated (co-IPed) with CDCA7_WT protein (peptide number ≥ 1.0), which were identified during our previous immunoprecipitation (IP)-MS/MS analysis^13^. A total of 42 proteins overlapped between the two sets (Fig. 1d and Supplementary Table S4). Notably, these 42 proteins included UHRF1 and two components of the facilitates chromatin transcription (FACT) complex, SUPT16H and structure specific recognition protein 1 (SSRP1).

To reduce the risk of false positives, we repeated our analysis with a more strict set of criteria and compared the 183 proteins identified by iPOND–MS/MS (≤ 0.5) with 29 proteins that co-IPed with CDCA7_WT protein (peptide number ≥ 5.0). This rigorous examination revealed seven overlapping proteins (Fig. 1e): DDX21, Ku80, SUPT16H, protein kinase DNA-activated catalytic subunit (PRKDC), histones H4 and H2B, and chromatin SWI/SNF-related matrix-associated actin-dependent regulator of chromatin subfamily A member 5 (SMARCA5). Among these, Ku80 and PRKDC are involved in NHEJ; we previously reported that CDCA7 and HELLS facilitate NHEJ by recruiting Ku80 at DNA damage sites^13^. Therefore, we focused on DDX21 and the FACT complex, the former of which is involved in R-loop resolution and the latter of which prevents R-loop formation by organizing chromatin structure^19,20^. Notably, these proteins continued to interact with CDCA7 following treatment with benzonase nuclease, which cleaves DNA and RNA^13^. Since some aberrantly generated R-loops could result in double-strand breaks (DSBs)^21^ and such DSBs are repaired by transcription-associated HR^22^, which accompanies DNA synthesis, both DDX21 and the FACT complex may work closely with proteins involved in the sequential process rather than those that are involved in DNA replication.

### Aberrant transcription and formation of DNA:RNA hybrids at pericentromeric repeats in ICF mutant cells

**Figure 2:**
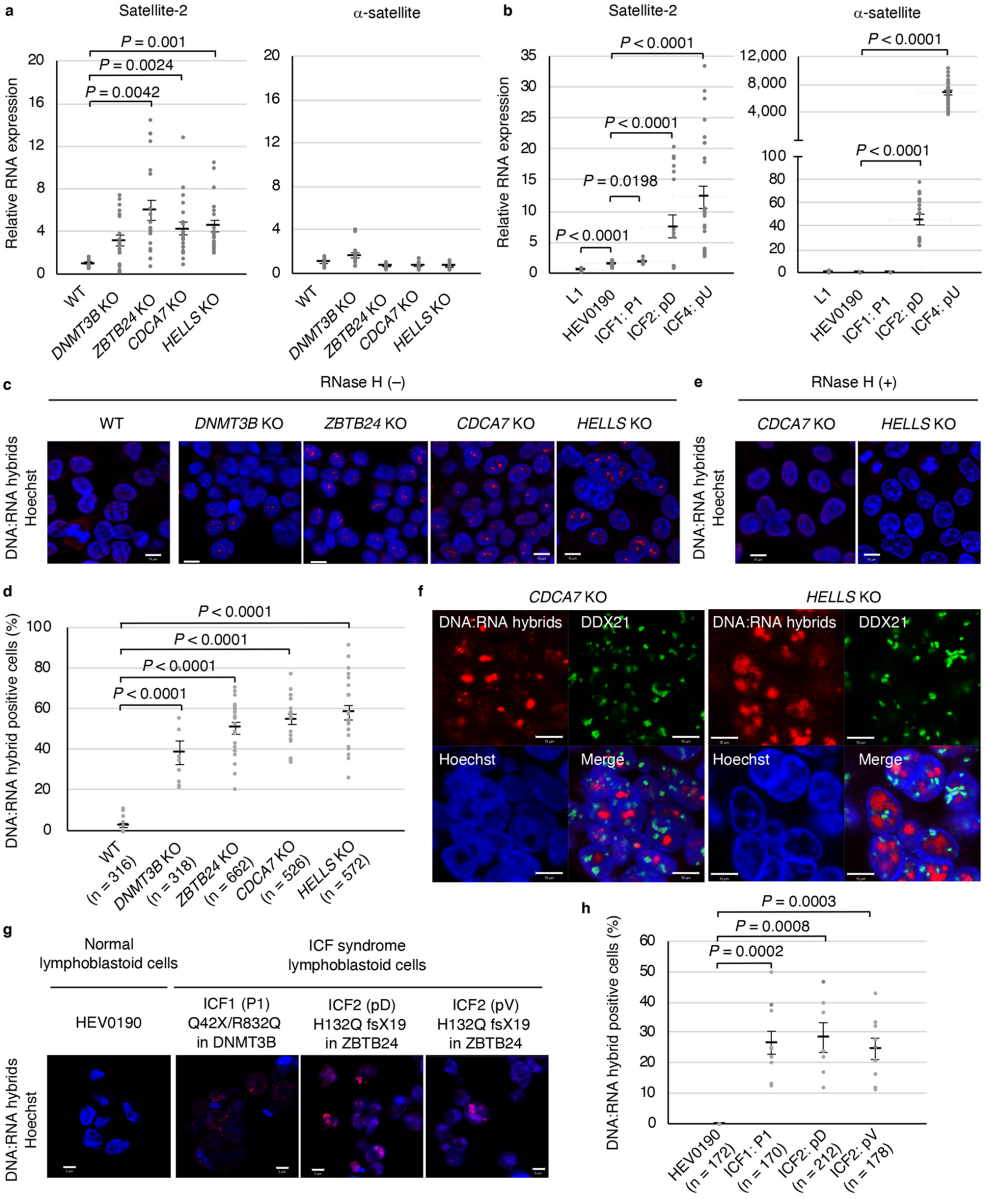
Aberrant transcription and formation of DNA:RNA hybrids in ICF mutant cells. (**a**) The relative expression of pericentromeric satellite-2 repeats and centromeric α-satellite repeats in WT, *DNMT3B* KO, *ZBTB24* KO, *CDCA7* KO, and *HELLS* KO HEK293 cells was examined by RT-qPCR; 18S ribosome RNA was used for normalization. No amplification of RT(–) samples was detected. Experiments were conducted in biological triplicate and technical triplicate. (**b**) The relative expression of satellite-2 repeats and α-satellite repeats in lymphoblastoid cells from two healthy subjects (L1 and HEV0190), an ICF1 patient (P1), an ICF2 patient (pD), and an ICF4 patient (pU) was examined by RT-qPCR; 18S ribosome RNA was used for normalization. No amplification of RT(–) samples was detected. Experiments were conducted in biological triplicate and technical triplicate. *P* values were calculated by comparison with HEV0190. (**c**) Representative images of DNA:RNA hybrids in WT and ICF mutant HEK293 cells detected using the S9.6 antibody. Hoechst 33,342 was used for DNA visualization (**c**,**e**,**f**,**g**). Scale bars: 10 µm (**c**,**e**,**f**). Experiments were conducted in biological triplicate and more than 300 cells were observed per each sample. (**d**) Summary of data shown in (**c**). The total cell number examined (n) is shown in parentheses (each dot represents one image) (**d,h**). (**e**) Representative images of DNA:RNA hybrids in *CDCA7* KO and *HELLS* KO HEK293 cells detected using S9.6 antibody with in vitro* E. coli* RNase H treatment. (**f**) DNA:RNA hybrids detected by the S9.6 antibody (red) were co-stained with DDX21 (green, a nucleoli marker) in *CDCA7* KO and *HELLS* KO cells (for other cells, see Supplementary Fig. S2). (**g**) Representative images of DNA:RNA hybrids in lymphoblastoid cells from a healthy female (HEV0190), an ICF1 patient (P1), and two ICF2 siblings (pD and pV) detected using the S9.6 antibody. Scale bars: 5 µm. More than 170 cells were observed per each sample. (**h**) Summary of data shown in (**g**). Data are mean ± s.e., and *P* values were obtained using the Mann–Whitney two-tailed *U* test (**a**,**b**,**d**,**h**).

It is well investigated that centromeric and pericentromeric repeats are hypomethylated in ICF patients^1^. Accordingly, we have previously observed extreme hypomethylation in pericentromeric satellite-2 repeats (*P* < 0.0004) and moderate hypomethylation (*P* ≤ 0.01) in centromeric α-satellite repeats in *ZBTB24* KO, *CDCA7* KO, and *HELLS* KO cells^13^. However, moderate hypomethylation of satellite-2 and no hypomethylation of α-satellite repeats occurred in *DNMT3B* KO cells^13^. Consistent with these observations, transcription from satellite-2 repeats was significantly higher in *ZBTB24* KO, *CDCA7* KO, and *HELLS* KO cells (*P* ≤ 0.01), whereas it was only moderately higher in *DNMT3B* KO cells (*P* > 0.05) (Fig. 2a). In contrast, transcription from the α-satellite repeats was not higher in any of the ICF mutant cells (Fig. 2a). We confirmed the result using lymphoblastoid cells from two healthy subjects (L1 and HEV0190), an ICF1 patient (P1) with heterozygous *DNMT3B* mutations (p.Q42X/p.R832Q), an ICF2 patient (pD) homozygous for a *ZBTB24* mutation (p.H132Q fsX19), and an ICF4 patient (pU) homozygous for a *HELLS* mutation (p.L801del)^13,23–25^ (Fig. 2b)*.* Although it was moderate, the expression from satellite-2 was increased in the ICF1 patient cells (*P* = 0.0198). Alternatively, transcription from both the satellite-2 and α-satellite repeats was significantly higher in the ICF2 and ICF4 patients’ cells (*P* < 0.0001) (Fig. 2b). The difference in the expression of α-satellite repeats between ICF mutant HEK293 cells and lymphoblastoid cells from the ICF2 and ICF4 patients could be due to different reduction levels of DNA methylation in the repeats.

The disruption of heterochromatin, which is comprised of repetitive elements, in the nematode *Caenorhabditis elegans* (*C. elegans*) triggers aberrant transcription, which results in DNA:RNA hybrid-associated repeat instability^26^. Therefore, we asked whether the hypomethylation of pericentromeric repeats in ICF cells could also give rise to the formation of aberrant DNA:RNA hybrids, leading to genome instability. We applied the widely used S9.6 antibody^27^ to detect DNA:RNA hybrids by immunofluorescence. DNA:RNA hybrids were significantly higher in all ICF mutant cells (Fig. 2c,d). We confirmed the specificity of the S9.6 antibody to DNA:RNA hybrids through the in vitro treatment of *Escherichia coli *(*E. coli*) RNase H (Fig. 2e). Intriguingly, in contrast to the results from many reports showing the accumulation of DNA:RNA hybrids in the nucleoli^19,28,29^, we found that DNA:RNA hybrids did not accumulate in the nucleoli detected by DDX21, which mainly localizes in the nucleoli^19^, in ICF mutant cells (Fig. 2f and Supplementary Fig. S2). Instead, DNA:RNA hybrids accumulated in Hoechst poor regions of the mutant cells. Of note, in the mutant cells, there were many nucleolar signals showing granulated and/or elongated patterns, which might be associated with the frequent appearance of polyploid cells with amplified ribosomal DNA^13^. This result suggests a possibility that the CDCA7/HELLS complex suppresses the formation of DNA:RNA hybrids at genomic regions outside the nucleoli via interactions with maintenance DNA methylation machinery and/or R-loop resolution/prevention. Since DNA:RNA hybrids were not detected on metaphase chromosomes (Supplementary Fig. S3), the hybrids seemed to either be resolved or further converted into DSBs by metaphase. The accumulation of DNA:RNA hybrids was also observed in the lymphoblastoid cells of the ICF1 patient (P1) and ICF2 siblings (pD and pV). In contrast, no such increase was observed in the cells of the healthy subject (HEV0190) (Fig. 2g,h).

### Accumulation of DNA:RNA hybrids at pericentromeric repeats in ICF mutant cells

**Figure 3:**
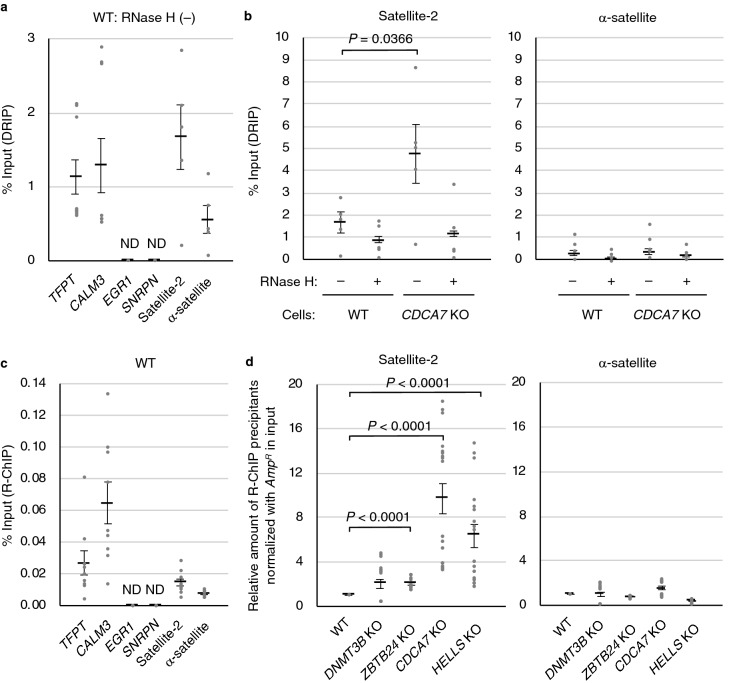
Aberrant accumulation of DNA:RNA hybrids at pericentromeric repeats in ICF mutant cells. (**a**) Validation of DRIP qPCR procedure. The efficiency of immunoprecipitation was validated with two previously known R-loop positive loci, *TFPT* and *CALM3*, and two negative loci, *EGR1* and *SNRPN*^30^, in RNase H untreated WT cells. The positive loci were successfully immunoprecipitated, whereas the Ct values of the negative loci were below the detection threshold. Amount of the immunoprecipitants was shown as percent input. ND, not detected. (**b**) Results of DRIP qPCR of satellite-2 and α-satellite in WT and *CDCA7* KO cells. DNA was extracted from these cells and treated with or without *E. coli* RNase H in vitro. Amount of the immunoprecipitants was shown as percent input. Experiments were conducted in biological duplicate and technical triplicate. (**c**) Validation of R-ChIP qPCR procedure. The efficiency of immunoprecipitation was validated with the two R-loop positive loci and two negative loci in WT cells. The positive loci were successfully immunoprecipitated, whereas the Ct values of the negative loci were below the detection threshold. Amount of the immunoprecipitants was shown as percent input. ND, not detected. (**d**) The V5–RNASEH1_D210N expressing plasmid vector was transfected with WT, *DNMT3B* KO, *ZBTB24* KO, *CDCA7* KO, and *HELLS* KO HEK293 cells, and R-ChIP qPCR was conducted using anti-V5 antibody 48 h after transfection. Relative amounts of immunoprecipitants with anti-V5 antibody were detected using primers for amplifying satellite-2 and α-satellite. Since transfection efficiency of the plasmid vector could be different between the cells, the *ampicillin-resistant* gene (*Amp*^*R*^) in the vector in input was used for normalization. The amount of immunoprecipitants of WT was considered to be 1.0. Experiments were conducted in biological triplicate and technical triplicate. Data are mean ± s.e. *P* values were obtained using the Mann–Whitney two-tailed *U* test.

To investigate whether aberrant DNA:RNA hybrids are formed at pericentromeric repeats, we conducted DNA-RNA immunoprecipitation (DRIP) analysis using genomic DNA extracted from WT and *CDCA7* KO cells and the S9.6 antibody. The efficiency of immunoprecipitation by the antibody was validated with two previously known R-loop positive loci, *TCF3 fusion partner* (*TFPT*) and *calmodulin 3* (*CALM3*), and two negative loci, *early growth response 1* (*EGR1*) and *small nuclear ribonucleoprotein polypeptide N* (*SNRPN*)^30^, in *E. coli* RNase H untreated WT cells. The positive loci were successfully immunoprecipitated, whereas the Ct values of the negative loci were below the detection threshold (Fig. 3a). In *CDCA7* KO cells, DNA:RNA hybrids significantly accumulated at pericentromeric satellite-2 repeats (*P* = 0.0366) but not at centromeric α-satellite repeats (Fig. 3b). The accumulation of hybrids at satellite-2 repeats in *CDCA7* KO cells was 4.2-fold and 3.7-fold higher than that at the *TPFT* and *CALM3* loci in WT cells on average, respectively (Fig. 3a,b).

The accumulation of DNA:RNA hybrids at satellite-2 in ICF mutant cells was further confirmed by R-loop chromatin immunoprecipitation (R-ChIP) analysis using an enzymatically dead V5-tagged human RNASEH1_D210N mutant, which stably localizes to DNA:RNA hybrids^31^. We transiently overexpressed the mutant RNASEH1 in WT and ICF mutant cells and conducted ChIP-qPCR. The efficiency of immunoprecipitation was validated again with the two R-loop positive loci and two negative loci by comparison with satellite-2 and α-satellite in WT cells. DNA:RNA hybrids accumulated at the *TFPT* and *CALM3* loci 1.8-fold and 4.4-fold more than those at satellite-2 in WT cells on average, whereas the Ct values of the negative loci were below the detection threshold (Fig. 3c). Because the transfection efficiency of the plasmid could be different among the WT and ICF mutant cells, we normalized amount of immunoprecipitants with the *ampicillin-resistance* genes (*Amp*^*R*^) in the plasmid in the input. In *CDCA7* KO and *HELLS* KO cells, DNA:RNA hybrids accumulated at pericentromeric satellite-2 repeats 9.7-fold and 6.3-fold more than those in WT cells on average (Fig. 3d). This result indicates that the hybrids accumulated at satellite-2 in *CDCA7* KO and *HELLS* KO cells more than those at the two R-loop positive loci in WT cells (Fig. 3c,d). The accumulation of DNA:RNA hybrids at centromeric α-satellite repeats was not observed in any ICF mutant cells (Fig. 3d). In *ZBTB24* KO cells, the accumulation of DNA:RNA hybrids at satellite-2 was observed but appeared moderate compared with the accumulation in *CDCA7* KO cells even though ZBTB24 is a transcriptional regulator of *CDCA7*^11^. This result may be due to the low-level expression of CDCA7 protein in *ZBTB24* KO cells^13^.

### Aberrant accumulation of DNA:RNA hybrids could trigger DNA damage in ICF mutant cells

**Figure 4:**
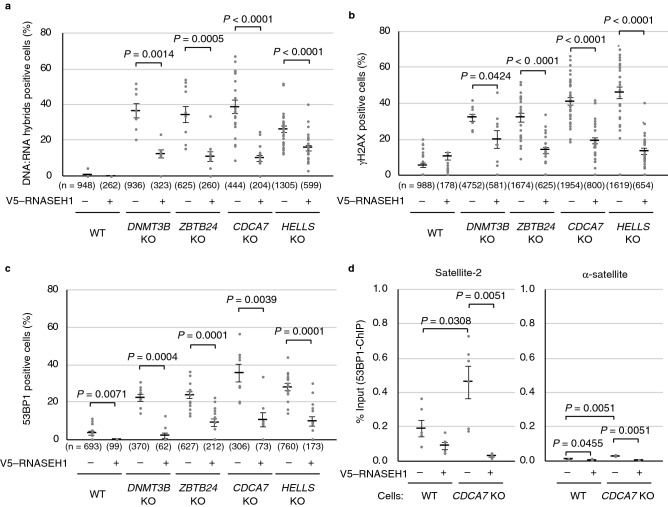
Accumulation of DNA:RNA hybrids in ICF mutant cells is a possible cause of DNA damage. (**a**) V5–RNASEH1_WT was transiently expressed in WT and ICF mutant cells; RNASEH1 was detected by anti-V5 antibody in green and DNA:RNA hybrids were detected by the S9.6 antibody in red (see Supplementary Fig. S6a). DNA:RNA hybrids positive cells in V5-RNASEH1_WT unexpressed (–) and expressed (+) cells were separately counted. Experiments were conducted in biological triplicate, and the total cell number examined (n) in more than ten images is shown in parentheses (each dot represents one image) (**a**–**c**). Data are mean ± s.e. (**a**–**c**). *P* values were obtained using the Mann–Whitney two-tailed *U* test (**a**–**c**). (**b**) V5–RNASEH1_WT was transiently expressed in WT and ICF mutant cells; RNASEH1 was detected by anti-V5 antibody in red and γH2AX foci were detected in green (see Supplementary Fig. S6b). γH2AX positive cells in V5-RNASEH1_WT unexpressed (–) and expressed (+) cells were separately counted. (**c**) V5–RNASEH1_WT was transiently expressed in WT and ICF mutant cells; RNASEH1 was detected by anti-V5 antibody in red and 53BP1 foci were detected in green (see Supplementary Fig. S6c). 53BP1 positive cells in V5-RNASEH1_WT unexpressed (–) and expressed (+) cells were separately counted. (**d**) Chromatin immunoprecipitation (ChIP) assay using anti-53BP1 antibody was conducted. WT and *CDCA7* KO cells with or without transfection of the V5–RNASEH1_WT were served for the assay. Satellite-2 repeats, α-satellite repeats, two R-loop positive loci (*TFPT* and *CALM3*), and two negative loci, (*EGR1* and *SNRPN*)^30^, were amplified. Ct values of the two positive and negative loci were below the detection threshold. Amount of immunoprecipitated fragments was calculated as percent input. Experiments were conducted in biological duplicate and technical triplicate.

Finally, we investigated whether the accumulation of DNA:RNA hybrids generated DSBs that we previously detected as the accumulation of γH2AX signals in ICF mutant cells^13^. Since γH2AX accumulation does not necessarily reflect the presence of DSBs^32^, we confirmed our previous data with the immunofluorescence of p53-binding protein 1 (53BP1), which is a key component of DSB signaling and repair in mammals^33^ (Supplementary Fig. S4). In human cells, there are two RNASEH proteins: H1 and H2^34^. It has been reported that H2 removes DNA:RNA hybrids in ribonucleoside monophosphates (rNMPs), R-loops, and possibly RNA primers at Okazaki fragments. H1 primarily resolves R-loops but does not induce unwanted nicks in the DNA at rNMPs, which could otherwise cause excessive DSB formation^34^. Therefore, we overexpressed V5-tagged human RNASEH1_WT to determine whether the removal of DNA:RNA hybrids could reduce DSBs in ICF mutant cells. The transient overexpression of V5–RNASEH1_WT itself was not toxic to the cells confirmed by flow cytometry (Supplementary Fig. S5). Strikingly, the overexpression of RNASEH1 reduced DNA:RNA hybrids detected by the S9.6 antibody (Fig. 4a and Supplementary Fig. S6a) and also reduced the signals of two DSB markers, γH2AX (Fig. 4b and Supplementary Fig. S6b) and 53BP1 (Fig. 4c and Supplementary Fig. S6c). Importantly, a ChIP assay using the anti-53BP1 antibody revealed that 53BP1 had significantly accumulated at satellite-2 repeats in *CDCA7* KO cells (*P* = 0.0308) and the accumulation was reduced by RNASEH1 expression (*P* = 0.0051) (Fig. 4d). No such 53BP1 accumulation at the two R-loop positive loci, *TFPT* and *CALM3*, and the two negative loci, *EGR1* and *SNRPN*, was observed; the Ct values of these loci were below the detection threshold. These data suggest that aberrant DNA:RNA hybrid formation is at least one of the causes of DSBs in these cells, although DSBs in ICF mutant cells may be reduced by RNASEH1, which could also rescue replication forks experiencing stress or reduce transcription.

## Discussion

ICF patients’ cells exhibit the disruption of pericentromeric heterochromatin composed of satellite-2 or satellite-3 repeats accompanied by DNA hypomethylation^1,13^. Our results suggest that the hypomethylation induces aberrant transcription and the formation of DNA:RNA hybrids (R-loops) at the repeats (Figs. 2, 3, 4), in a manner reminiscent of the *C. elegans met-2 set-25* double mutant, which exhibits repeat instability due to the lack of H3K9 methylation^26^*.* The DNA hypomethylation could be due to inefficient recruitment of the DNMT1/UHRF1 maintenance DNA methylation machinery at heterochromatic and late replicating regions^15,16^ in ICF cells (Fig. 1). Such heterochromatic and late replicating regions may depend on the dual monoubiquitination of histone H3 via UHRF1 for proper maintenance DNA methylation^16^. It has been shown that hemimethylated DNA wrapped around nucleosomes is not a preferred substrate of DNMT1^17^, and mouse and plant HELLS homologs facilitate methylation of DNA wrapped around nucleosomes in vivo^35^. Therefore, chromatin remodeling by the CDCA7/HELLS complex at heterochromatic and late replicating regions might be required for full methylation by DNMT1. This hypothesis can explain why these regions are particularly vulnerable to DNA damage in ICF cells.

The machinery that resolves or prevents the formation of aberrant DNA:RNA hybrids could be impaired in ICF mutant cells, or the excessive formation of aberrant DNA:RNA hybrids at hypomethylated regions in ICF mutant cells could exceed the capacity of the machinery, which could lead to DSBs (Fig. 4). DDX21 and the FACT complex, both of which interact with CDCA7, could be important components of this machinery, but further examination is needed to reveal the details of this theory.

Since NHEJ is defective in ICF mutant cells^13^ and R-loop-associated DNA damage is preferentially repaired by transcription-associated HR^22^, centromeric instability represented by multiradial chromosomes observed in ICF cells could result from unresolved Holliday junctions generated by aberrant HR between satellite-2 repeats in different chromosomes^2,36^. We propose that pericentromeric stability is protected by multiple molecular layers, including DNA methylation, R-loop resolution/prevention, and NHEJ by the CDCA7/HELLS chromatin remodeling complex and/or DNMT3B, and that the disruption of these protections could cause specific ICF syndrome phenotypes.

## Methods

### Cells

A lymphoblastoid cell line from a healthy female subject (HEV0190) was purchased from the RIKEN Cell Bank (Tsukuba, Japan)^25^, and a line from an ICF1 patient (P1) was acquired from a cell bank maintained in the Saitama Children’s Medical Center^23^. Lymphoblastoid cell lines of the healthy male subject (L1), two ICF2 siblings (pD and pV), and an ICF4 patient (pU) were previously established with informed consent in our lab^10,15,24^. The lymphoblastoid cells were maintained in RPMI 1640 GlutaMAX medium supplemented with 20% fetal bovine serum (FBS) and penicillin–streptomycin at 37 °C and 5% CO_2_. HEK293 cells were attained from the American Type Culture Collection (Gaithersburg, MD, USA) and maintained in Dulbecco's Modified Eagle's Medium (Nacalai tesque, Kyoto, Japan) supplemented with 10% FBS and penicillin–streptomycin at 37 °C and 5% CO_2_. The *DNMT3B* KO, *ZBTB24* KO, *CDCA7* KO, and *HELLS* KO HEK293 cells were previously generated using a CRISPR/Cas9 system^13^.

### Plasmids and antibodies

The ppyCAG_RNASEH1_WT and D210N plasmids encoding V5-tagged proteins were gifts from Xiang-Dong Fu (Addgene plasmid numbers 111906 and 111904)^31^. The antibodies used in the present study are summarized in Supplementary Table S5.

### iPOND

iPOND analysis was conducted as previously described with minor modifications^37^. Briefly, WT and *CDCA7* KO cells were plated the day before the experiment. On the following day, EdU was added to the medium at a final concentration of 10 µM and incubated for 20 min. Afterward, the cells were harvested, washed with DMEM, and cross-linked with 1% formaldehyde at room temperature for 10 min. Fixation was stopped by the addition of 125 mM glycine and incubating at room temperature for 5 min. The cells were washed with phosphate-buffered saline (PBS) and permeabilized with 0.25% Triton X-100 in PBS at room temperature for 30 min. After washing the cells with 0.25% bovine serum albumin (BSA) in PBS, a click chemistry reaction was conducted using a click reaction cocktail (10 mM sodium ascorbate, 2 mM CuSO_4_, and 10 μM biotin-azide, in PBS) at room temperature for 1.5 h. The cells were lysed in a lysis buffer (1% sodium dodecyl sulfate [SDS] in 50 mM Tris-HCl, pH 8.0), and chromatin DNA was sheared to approximately 800 bp fragments by sonication. The supernatant was diluted twice with the same amount of PBS; 5% of it was saved as input, and the remaining supernatant was rotated with streptavidin agarose (Millipore, Burlington, MA, USA) at 4 °C overnight. The streptavidin agarose containing the captured DNA–protein complexes was washed once with lysis buffer and five times with 1 M NaCl. The DNA–protein complex was eluted with Laemmli Sample buffer (Bio-Rad, Hercules, CA, USA) for Western blotting or sodium deoxycholate (SDC) elution buffer (24 mM SDC and 24 mM sodium lauroyl sarcosinate in 500 mM Tris–HCl pH 8.0) for MS/MS analysis, and denatured at 95 °C for 20 min to 1 h.

### Reverse transcription (RT)-quantitative PCR (qPCR)

Total RNAs were extracted from cells using ISOGEN (Nippon Gene, Tokyo, Japan). cDNAs were generated using a PrimeScript RT reagent Kit with gDNA Eraser (TaKaRa Bio, Shiga, Japan), with [RT(+)] or without [RT(–)] PrimeScript enzyme Mix I, according to the manufacturers’ protocols. The primers used for RT-qPCR are summarized in Supplementary Table S6. PCR reactions were conducted using a Thermal Cycler Dice Real Time System Single (TaKaRa Bio), according to the manufacturer’s protocol. The samples were amplified with the following program: one cycle of 95 °C for 30 s, followed by 40 cycles of 95 °C for 5 s and 60 °C for 30 s. The 18S ribosomal RNA levels were used for normalization. The relative RNA abundance of each gene or genomic region in ICF mutant cells was calculated by comparison with that of WT cells. The Ct values of all the RT(–) samples were below the detection threshold.

### Immunofluorescence

The immunofluorescence staining of DNA:RNA hybrids using the S9.6 antibody and anti-53BP1 antibody (combination with anti-V5 or anti-γH2AX antibodies) was conducted as previously described with minor modifications^38,39^. Adherent cells were seeded in chamber slides the day before the immunofluorescence experiments, and the cells were fixed with ice-cold methanol for 10 min. Lymphoblastoid cells were fixed with ice-cold methanol for 10 min, spread on a glass slide, and dried on a heat block at 50 °C. Following methanol fixation, cells were permeabilized with acetone for 1 min (for the S9.6 antibody only), washed with PBS three times, and blocked with 3% BSA and 0.1% Tween 20 in 4 × saline sodium citrate (SSC) buffer (600 mM sodium chloride and 60 mM trisodium citrate, pH 7.0). If indicated, the slides were treated with 120U of *E. coli* RNase H (TaKaRa Bio, catalog 2150A) in buffer (40 mM Tris-HCl pH8.0, 4 mM MgCl_2_, 1 mM dithiothreitol, 4% glycerol, and 0.003% BSA) at 37 °C for 4 h; in the case, the RNase H (–) cells were treated in the exact same manner as the RNase H ( +) cells except the enzyme. The cells were then incubated with S9.6 antibodies (1:100) in 4 × SSC with 3% BSA and 0.1% Tween 20 for 2 h to overnight. After washing three times with 4 × SSC, the cells were incubated with Alexa Fluor 594 donkey anti-mouse IgG H&L antibody (Abcam, UK, Cambridge, catalog 150112, 1:1000) in 4 × SSC with 3% BSA and 0.1% Tween 20 for 1 h at room temperature. The nuclei were visualized using Hoechst 33342 (PromCell, Heidelberg, Germany). Fluorescence images were taken using an LSM700 confocal laser scanning microscope (Carl Zeiss, Jena, Germany).

Immunofluorescence of the V5 tag and γH2AX was conducted as previously described with minor modifications^13^. Briefly, the cells were transfected with ppyCAG_RNASEH1_WT (V5 tag) using FuGene HD transfection reagent (Promega, Madison, WI, USA). The cells were fixed 48 h after transfection with 4% paraformaldehyde in PBS for 30 min, permeabilized with 0.5% Triton X-100 in PBS for 30 min, and blocked with Block Ace (DS Pharma Biomedical, Osaka, Japan) for 1 h at room temperature. Cells were incubated with the anti-V5 and anti-γH2AX antibodies at room temperature for 1 h. After washing with PBS, cells were incubated with Alexa Fluor 594 donkey anti-mouse IgG H&L antibody (Abcam, catalog 150112, 1:1000) and Alexa Fluor 488 donkey anti-rabbit IgG H&L antibody (Abcam, catalog 150061, 1:1000) at room temperature for 1 h. The nuclei were visualized using Hoechst 33342. Fluorescence images were taken using an LSM700 confocal laser scanning microscope (Carl Zeiss).

### DRIP qPCR

A DRIP assay was conducted as previously described with minor modifications^20^. Briefly, purified DNA, including DNA:RNA hybrids, extracted from WT and *CDCA7* KO HEK293 cells was digested with a cocktail of restriction enzymes (ApaLI, NotI, SspI, EcoRI, and HindIII) in the absence or presence of *E. coli* RNase H (TaKaRa Bio) at 37 °C overnight. Then, the DNA was further sonicated and electrophoresed using an agarose gel; 100 bp–1.4 kb fragments were cut off from the gel and extracted using a QIAquick gel extraction kit (QIAGEN, Hilden, Germany). The extracted fragments diluted in binding buffer (10 mM NaPO_4_ pH 7.0, 140 mM NaCl, and 0.05% TritonX-100) were immunoprecipitated with the S9.6 antibody at 4 °C overnight. Protein A/G PLUS–Agarose (Santa Cruz Biotechnology, Dallas, TX, USA) was added and rotated at 4 °C for 2 h. After washing the agarose five times with a lysis buffer, the S9.6 antibody was digested by proteinase K (Merck, Kenilworth, NJ, USA) in elution buffer (50 mM Tris–HCl pH 8.0, 10 mM ethylenediaminetetraacetic acid [EDTA], and 0.5% SDS] at 50 °C for 1 h. The nucleic acids were collected by standard ethanol precipitation and eluted in H_2_O. qPCR was conducted using the primers indicated in Supplementary Table S6. Input was used for the normalization of each sample, and amount of immunoprecipitated fragments was calculated as percent input.

### R-ChIP and ChIP qPCR

The R-ChIP and standard ChIP assays were conducted as previously described with minor modifications^31^. Briefly, cells were transfected with ppyCAG_RNASEH1_D210N harboring the *Amp*^*R*^ gene (R-ChIP) or ppyCAG_RNASEH1_WT (ChIP). The cells were cross-linked 48 h after transfection with 1% formaldehyde at room temperature for 15 min. Fixation was stopped by the addition of glycine to a final concentration of 125 mM and incubation at room temperature for 15 min. After washing the plates twice with PBS, the cells were scraped off, and the nuclei were extracted with cell lysis buffer (10 mM Tris-HCl pH 8.0, 1 mM NaCl, 0.5% NP-40, and protease inhibitor cocktail). The cells were suspended in nuclei lysis buffer (50 mM Tris–HCl pH 8.0, 10 mM EDTA, 1% SDS, and protease inhibitor cocktail). Chromatin DNA was sheared into fragments of approximately 800 bp by sonication. 5% of the chromatin fragments were saved as input, and the remaining fraction was rotated with the anti-V5 antibody (R-ChIP) or anti-53BP1 antibody (ChIP) at 4 °C for more than 3 h. Afterward, Protein A/G PLUS–agarose was added and rotated at 4 °C for 1–2 h. Immunoprecipitants were sequentially washed three times with TSEI (20 mM Tris-HCl pH 8.0, 150 mM NaCl, 1% Triton X-100, 0.1% SDS, and 2 mM EDTA), three times with TSEII (20 mM Tris-HCl pH 8.0, 500 mM NaCl, 1% Triton X-100, 0.1% SDS, and 2 mM EDTA), once with TSEIII (10 mM Tris-HCl pH 8.0, 250 mM LiCl, 1% NP-40, 1% deoxycholate, and 1 mM EDTA), and once with TE buffer (10 mM Tris-HCl pH 8.0, and 1 mM EDTA). The protein–chromatin complex was eluted with elution buffer (10 mM Tris-HCl pH 8.0, 1% SDS, and 1 mM EDTA) and de-crosslinked by incubation at 65 °C for more than 2 h. After sequential treatment with RNase A (Roche, Basel, Switzerland) at 37 °C for 1 h and proteinase K (Merck) at 50 °C for more than 2 h, the precipitated hybrid fragments were cleaned with the QIAquick PCR purification kit (QIAGEN) or phenol–chloroform extraction with ethanol precipitation. The recovered fragments were subjected to qPCR. The primers used for qPCR are summarized in Supplementary Table S6. PCR reactions were conducted using a Thermal Cycler Dice Real Time System Single (TaKaRa Bio) according to the manufacturer’s protocol. The samples were amplified with the following program: one cycle of 95 °C for 30 s, followed by 40 cycles of 95 °C for 5 s and 60 °C for 30 s. Percent input of immunoprecipitants was calculated for two R-loop positive and two negative loci, satellite-2, and α-satellite in WT cells (for R-ChIP) or WT and *CDCA7* KO cells (for ChIP). If indicated, the *ampicillin-resistant* gene (*Amp*^*R*^) of the plasmid vector in “input” was used for the normalization of each sample. The relative abundance of each genomic region in ICF mutant cells was calculated by comparison with WT cells as 1.0 (R-ChIP).

### Statistics

The Mann–Whitney two-tailed *U* test was conducted for the statistical analysis of all data presented in graphs. All data are presented in graphs as the mean value ± standard error (s.e.).

### Study approval

All clinical samples were obtained in an anonymized manner and written informed consent was obtained from participants before their inclusion in the present study. The Kyushu University Institutional Review Board for Human Genome/Gene Research (#599-02) and the local ethics committee of Necker-Enfants Malades Hospital, Paris, France, approved the present study. All methods were conducted according to relevant guidelines and regulations.

## Supplementary information

Supplementary Information. (32324 kb)
