# Supplementary material for: CDCA7 and HELLS suppress DNA:RNA hybrid-associated DNA damage at pericentromeric repeats
Source: Sci Rep. 2020 Oct 20;10:17865. doi: 10.1038/s41598-020-74636-2 (PMC7576824; doi:10.1038/s41598-020-74636-2)

# Supplementary Information

## CDCA7 and HELLS suppress DNA:RNA hybrid-associated DNA damage at pericentromeric repeats

Motoko Unoki[1,*], Jafar Sharif[2], Yuichiro Saito[3], Guillaume Velasco[4], Claire Francastel[4], Haruhiko Koseki[2] & Hiroyuki Sasaki[1]

[1]Division of Epigenomics and Development, Medical Institute of Bioregulation, Kyushu University, Fukuoka, 812-8582, Japan.
[2]Laboratory for Developmental Genetics, RIKEN Center for Interactive Medical Sciences, Kanagawa,230-0045, Japan.
[3]Division of Molecular Cell Engineering, National Institute of Genetics, Research Organization of Information and Systems (ROIS), Mishima, Shizuoka, 411-8540, Japan.
[4]CNRS UMR7216, Epigenetics and Cell Fate, Université Paris Diderot, Sorbonne Paris Cité, 75205 Paris, France.

Correspondence
Motoko Unoki, Division of Epigenomics and Development, Medical Institute of Bioregulation, Kyushu University, 3-1-1 Maidashi, Higashi-ku, Fukuoka-shi, Fukuoka, 812-8582, Japan.
Phone: 81.92.642.6761, email: unokim@bioreg.kyushu-u.ac.jp

**Supplementary Methods**
**Supplementary Figures S1-S6**
**Supplementary Tables S1-S6**

## Supplementary Methods

**Flow cytometry.** Cells were transfected with or without ppyCAG_RNASEH1_WT (V5 tag) using FuGene HD transfection regent (Promega). The cells were fixed 48 h after transfection with 4% paraformaldehyde in PBS for 15 min, permeabilized with ice-cold methanol for 10 min, washed with antibody solution (1% BSA and 0.2% Tween 20 in PBS) two times, and blocked with 5% BSA in PBS at room temperature for 30 min. The cells were incubated with an anti-V5 antibody at room temperature for 2 h. After washing with antibody solution two times, the cells were incubated with Alexa Fluor 488 donkey anti-mouse IgG H&L antibody (Abcam, catalog ab150109, 1:1000) at room temperature for 1 h. After washing with antibody solution two times, DNA was stained by FxCycle Violet (Thermo Fisher) and subjected to flow cytometric analysis using FACSVerse (BD Biosciences). A 488 negative fraction of V5–RNASEH1_WT untransfected cells and a 488 positive fraction of V5–RNASEH1_WT transfected cells were gated and served for cell cycle analysis. The percentage of the nuclei in G0/G1, S, and G2/M phases of the cell cycle, and any sub-G1 population or polyploidy, were determined from the gated cells.

## Supplementary Figures

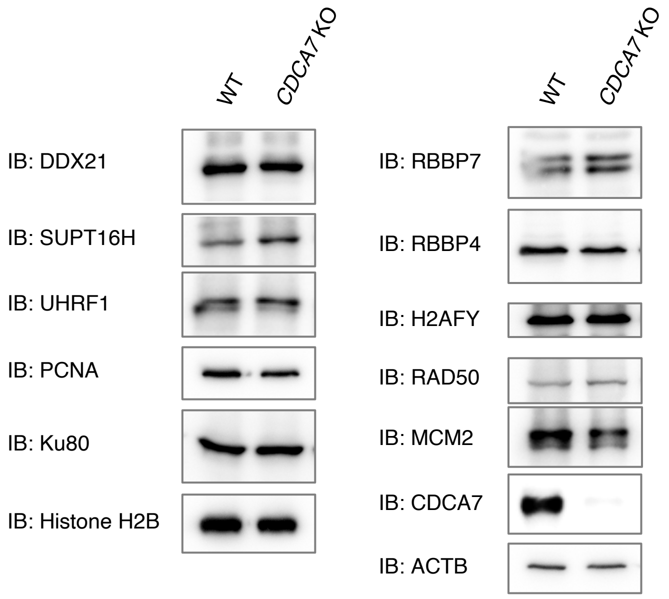


**Supplementary Figure S1.** Expression of representative proteins in WT and *CDCA7* KO HEK293 cells. Same amount of total cell lysate from WT and *CDCA7* KO cells was applied for each lane. Anti-CDCA7 antibody was used for confirming KO of CDCA7 and ACTB was used as a loading control.

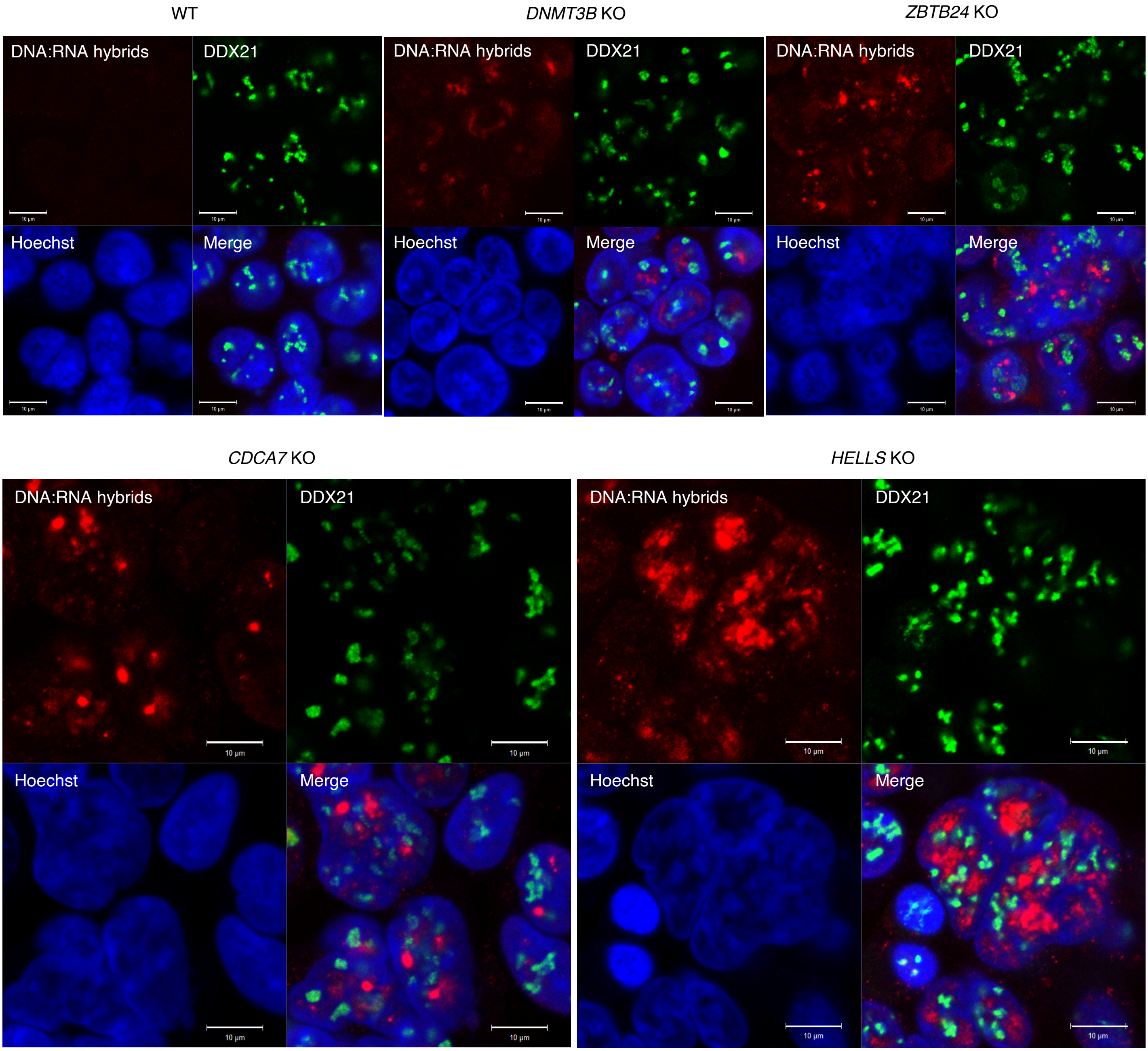


**Supplementary Figure S2.** Distinctive accumulation of DNA:RNA hybrids in ICF mutant cells. Wild-type (WT) and ICF mutant HEK293 cells were stained with the S9.6 antibody (red) and an anti-DDX21 antibody (green). DDX21 is known to mainly localize in the nucleoli. Hoechst 33342 was used for DNA visualization. DNA:RNA hybrids visualized by the S9.6 antibody were excluded from the nucleoli and accumulated at relatively Hoechst 33342 weak regions in ICF mutant cells. Scale bars: 10 µm.

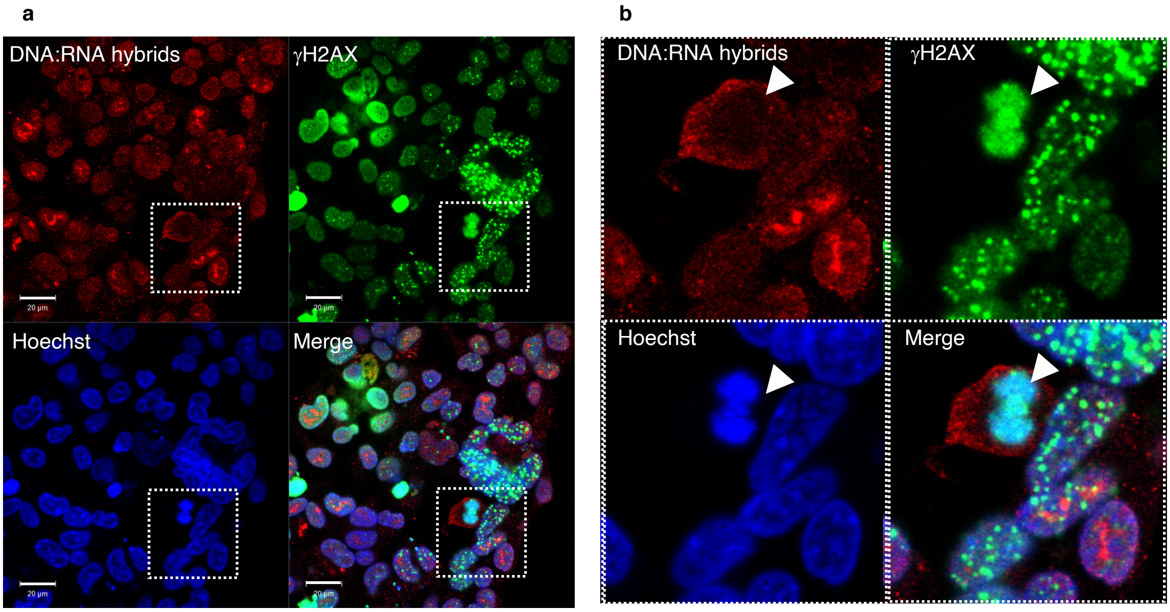


**Supplementary Figure S3.** DNA:RNA hybrids are not detected on metaphase chromosomes in ICF mutant cells. (**a**) A representative image of DNA:RNA hybrids (red) detected using the S9.6 antibody and $\gamma$H2AX (green) in *CDCA7* KO HEK293 cells. Hoechst 33342 was used for DNA visualization. Scale bars: 20 $\mu$m. DNA:RNA hybrids were not detected on metaphase chromosomes in any ICF mutant cells ($n \geq 50$). (**b**) An enlarged image of the regions indicated as boxes with broken line in (**a**). Arrowheads indicate metaphase chromosomes. In metaphase cells, relatively strong cytoplasmic signals were observed.

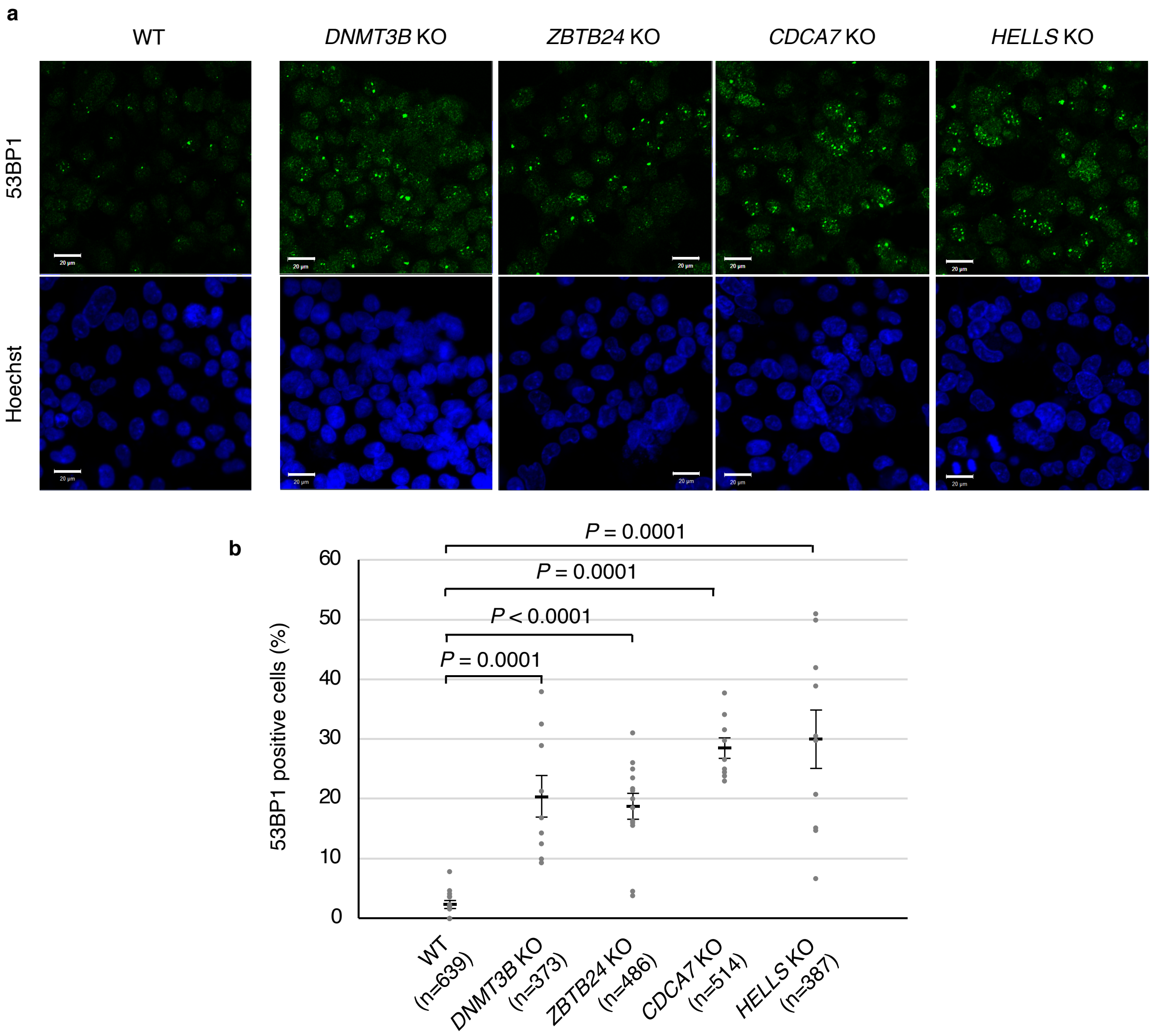


**Supplementary Figure S4.** 53BP1 is accumulated in ICF mutant cells. (**a**) Accumulation of DSBs in ICF mutant cells was confirmed by 53BP1 staining (green). Hoechst 33342 was used for DNA visualization. Scale bars: 20 μm. (**b**) Summary of data shown in (**a**). The total cell number examined (n) is shown in parentheses. Data are mean ± s.e. *P* values were obtained using the Mann–Whitney two-tailed *U* test.

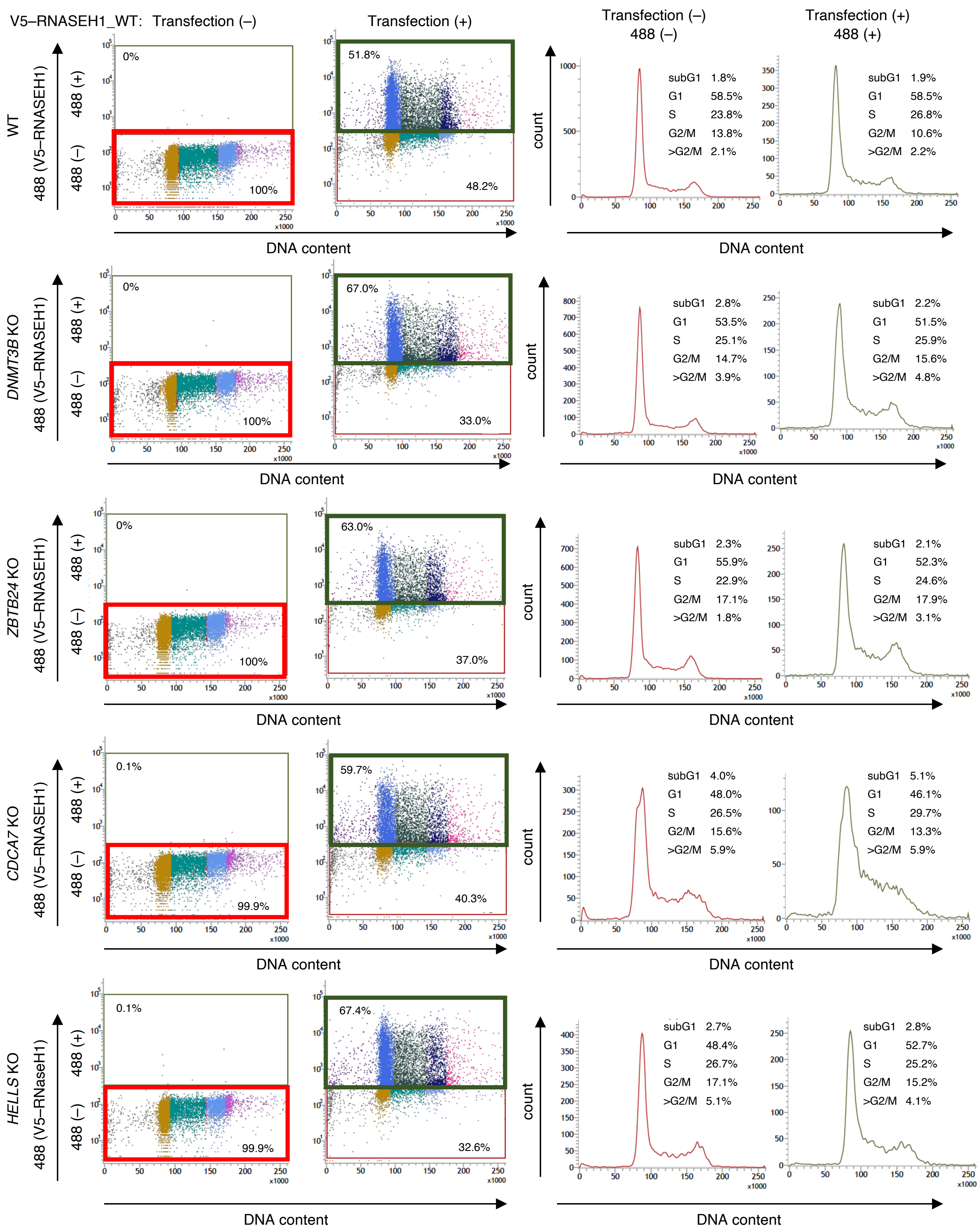


**Supplementary Figure S5.** Transient overexpression of V5–RNASEH1_WT does not affect cell cycle. Wild-type (WT) and ICF mutant cells were transfected with or without V5–RNASEH1_WT. After 48 h of transfection, cells were immunostained with an anti-V5 antibody (488 nm) and DNA was stained by FxCycle Violet. Then, a 488 (–) fraction of V5–RNASEH1_WT untransfected cells (red) and a 488 (+) fraction of V5–RNASEH1 WT transfected cells (green) were served for cell cycle analysis by flow cytometry. Cell population (%) in each cycle was indicated.

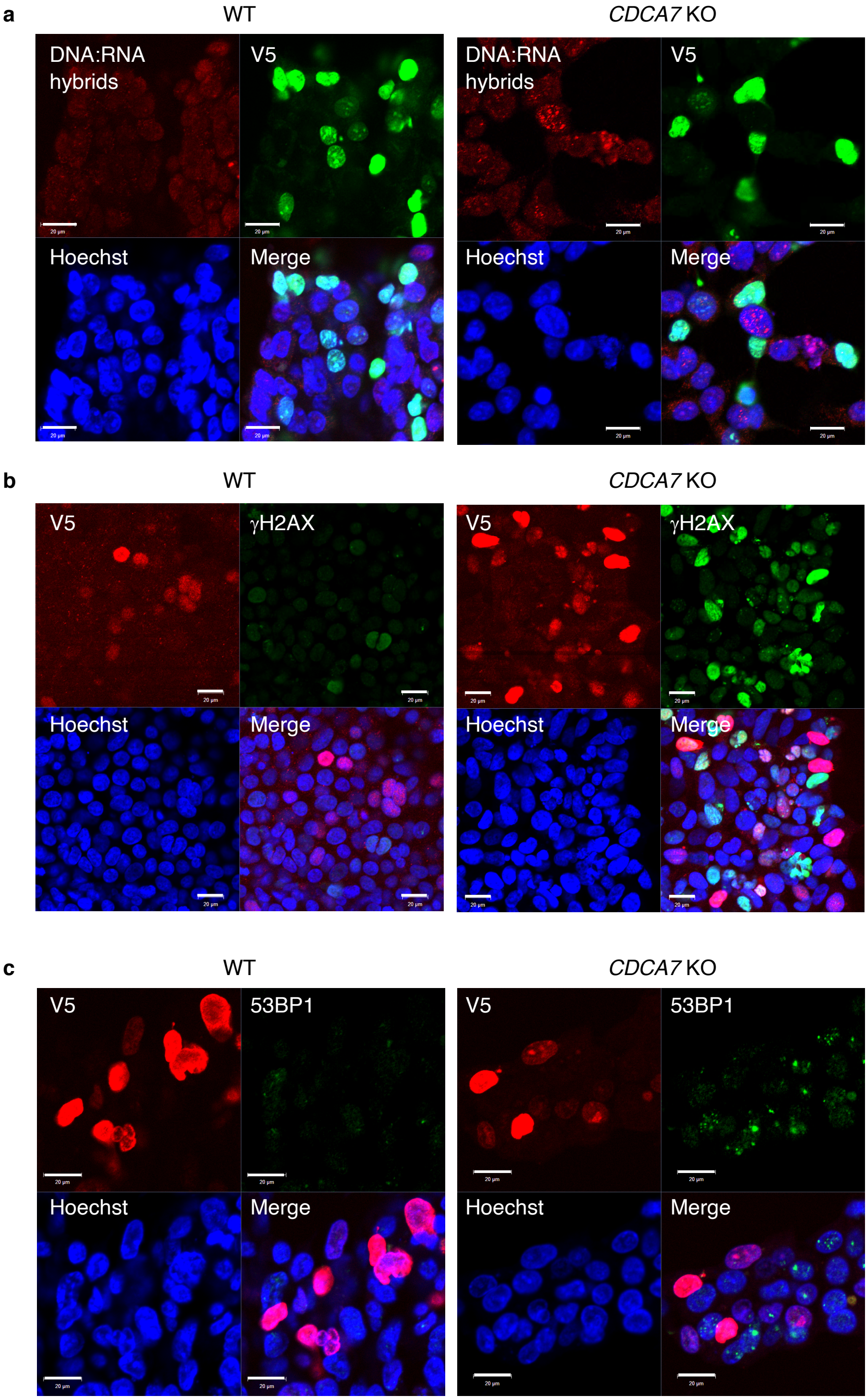


**Supplementary Figure S6.** RNASEH1 expression reduces DNA:RNA hybrids and double-strand breaks. (**a**) Representative images of immunofluorescence detecting exogenous expression of V5–RNASEH1_WT in green and DNA:RNA hybrids by the S9.6 antibody in red. (**b**) Representative images of immunofluorescence detecting exogenous expression of V5–RNASEH1_WT in red and γH2AX in green. (**c**) Representative images of immunofluorescence detecting exogenous expression of V5–RNASEH1_WT in red and 53BP1 in green. Hoechst 33342 was used for DNA visualization and scale bars represent 20 μm (**a**, **b**, **c**).

## Supplementary Tables

**Supplementary Table S1.** Proteins decreased on nascent DNA in *CDCA7* KO HEK293 cells determined by iPOND–MS/MS analysis (≤0.66). Related to Figure 1a.

| Protein | *WT (norm MS/MS score) | **CDCA7* KO (norm MS/MS score) | KO/WT | Protein | *WT (norm MS/MS score) | **CDCA7* KO (norm MS/MS score) | KO/WT |
|---|---|---|---|---|---|---|---|
| ATP5PD | 6.05.E-04 | 1.78.E-05 | 0.03 | SUMO1 | 2.25.E-03 | 6.76.E-04 | 0.30 |
| TPX2 | 5.04.E-04 | 1.71.E-05 | 0.03 | ANP32E | 3.83.E-04 | 1.15.E-04 | 0.30 |
| PABPC3 | 2.60.E-04 | 9.44.E-06 | 0.04 | PNN | 2.42.E-04 | 7.32.E-05 | 0.30 |
| THOC4 | 1.72.E-04 | 6.29.E-06 | 0.04 | RFC2 | 2.55.E-04 | 7.73.E-05 | 0.30 |
| CSNK2B | 5.29.E-04 | 2.15.E-05 | 0.04 | TIA1 | 3.39.E-05 | 1.03.E-05 | 0.30 |
| VRK1 | 2.58.E-04 | 1.14.E-05 | 0.04 | RPL13 | 4.73.E-04 | 1.47.E-04 | 0.31 |
| RPL18 | 2.54.E-04 | 1.58.E-05 | 0.06 | SF3A1 | 3.62.E-04 | 1.12.E-04 | 0.31 |
| SRSF5 | 1.91.E-04 | 1.26.E-05 | 0.07 | NIPBL | 7.68.E-05 | 2.38.E-05 | 0.31 |
| CSK | 1.77.E-04 | 1.47.E-05 | 0.08 | PHF8 | 1.02.E-04 | 3.18.E-05 | 0.31 |
| CNOT1 | 7.73.E-05 | 7.38.E-06 | 0.10 | EIF4A3 | 3.14.E-04 | 9.88.E-05 | 0.31 |
| TUBB4B | 2.19.E-04 | 2.12.E-05 | 0.10 | DDB1 | 3.38.E-04 | 1.07.E-04 | 0.32 |
| EIF4H | 3.53.E-04 | 3.83.E-05 | 0.11 | SRRT | 5.15.E-05 | 1.64.E-05 | 0.32 |
| XRCC1 | 1.58.E-04 | 1.71.E-05 | 0.11 | TXLNA | 1.43.E-04 | 4.56.E-05 | 0.32 |
| CDC37 | 2.61.E-04 | 3.11.E-05 | 0.12 | EIF3C | 1.25.E-04 | 4.02.E-05 | 0.32 |
| EIF3B | 5.63.E-04 | 7.05.E-05 | 0.13 | IK | 1.01.E-04 | 3.26.E-05 | 0.32 |
| ADNP | 1.05.E-03 | 1.33.E-04 | 0.13 | POLA1 | 1.01.E-04 | 3.29.E-05 | 0.32 |
| LUC7L2 | 1.95.E-04 | 2.63.E-05 | 0.13 | POLR2B | 1.51.E-04 | 5.00.E-05 | 0.33 |
| DDX23 | 1.02.E-04 | 1.40.E-05 | 0.14 | ILF2 | 2.34.E-03 | 7.86.E-04 | 0.34 |
| HMGN3 | 8.91.E-05 | 1.26.E-05 | 0.14 | CAPRIN1 | 2.04.E-04 | 6.95.E-05 | 0.34 |
| HIST3H3 | 7.15.E-04 | 1.02.E-04 | 0.14 | ILKAP | 2.80.E-04 | 9.54.E-05 | 0.34 |
| UBA1 | 1.09.E-04 | 1.55.E-05 | 0.14 | SSB | 2.92.E-04 | 1.01.E-04 | 0.35 |
| HDAC2 | 1.09.E-03 | 1.71.E-04 | 0.16 | TCP1 | 1.14.E-04 | 3.96.E-05 | 0.35 |
| DNAJC9 | 4.32.E-04 | 6.97.E-05 | 0.16 | SMCHD1 | 2.83.E-04 | 1.00.E-04 | 0.35 |
| GTF2I | 6.11.E-03 | 9.90.E-04 | 0.16 | MTA1 | 3.60.E-04 | 1.27.E-04 | 0.35 |
| PRPF3 | 1.03.E-04 | 1.69.E-05 | 0.16 | HMG20A | 6.58.E-04 | 2.34.E-04 | 0.35 |
| SMC2 | 2.25.E-04 | 3.75.E-05 | 0.17 | CBX3 | 4.56.E-03 | 1.62.E-03 | 0.36 |
| ADAR | 2.19.E-04 | 3.70.E-05 | 0.17 | EEF1E1 | 7.39.E-05 | 2.65.E-05 | 0.36 |
| DYNC1I2 | 7.21.E-05 | 1.23.E-05 | 0.17 | WDHD1 | 1.66.E-04 | 5.99.E-05 | 0.36 |
| RIF1 | 2.84.E-04 | 4.98.E-05 | 0.18 | RAD21 | 5.42.E-04 | 1.95.E-04 | 0.36 |
| THOC2 | 1.30.E-04 | 2.43.E-05 | 0.19 | SF3B3 | 1.94.E-04 | 7.03.E-05 | 0.36 |
| POLR2E | 1.62.E-04 | 3.10.E-05 | 0.19 | DEK | 4.93.E-03 | 1.80.E-03 | 0.36 |
| PRPF6 | 2.30.E-04 | 4.42.E-05 | 0.19 | NONO | 1.23.E-03 | 4.55.E-04 | 0.37 |
| DDX21 | 1.15.E-04 | 2.26.E-05 | 0.20 | ACTL6A | 4.09.E-04 | 1.53.E-04 | 0.37 |
| SUPT6H | 1.06.E-04 | 2.08.E-05 | 0.20 | MBD3 | 6.26.E-04 | 2.34.E-04 | 0.37 |
| RNPS1 | 1.42.E-04 | 2.82.E-05 | 0.20 | PDS5A | 7.98.E-04 | 3.01.E-04 | 0.38 |
| API5 | 1.96.E-04 | 4.01.E-05 | 0.20 | MTA2 | 6.37.E-04 | 2.41.E-04 | 0.38 |
| RECQL | 7.84.E-04 | 1.61.E-04 | 0.21 | HNRNPA3 | 4.16.E-03 | 1.58.E-03 | 0.38 |
| SRRM1 | 7.55.E-04 | 1.55.E-04 | 0.21 | HNRNPR | 1.88.E-03 | 7.15.E-04 | 0.38 |
| CBX5 | 5.53.E-04 | 1.20.E-04 | 0.22 | FKBP3 | 1.53.E-04 | 5.82.E-05 | 0.38 |
| TCOF1 | 1.21.E-04 | 2.83.E-05 | 0.23 | SNRPC | 1.38.E-04 | 5.24.E-05 | 0.38 |
| RPS3A | 8.88.E-04 | 2.10.E-04 | 0.24 | DNAJB1 | 1.47.E-04 | 5.60.E-05 | 0.38 |
| SF3B1 | 1.27.E-03 | 3.04.E-04 | 0.24 | TCEA1 | 2.72.E-04 | 1.04.E-04 | 0.38 |
| WIZ | 1.28.E-03 | 3.13.E-04 | 0.25 | PRPF8 | 1.16.E-03 | 4.45.E-04 | 0.38 |
| RPRD1B | 9.90.E-05 | 2.48.E-05 | 0.25 | TCERG1 | 3.50.E-04 | 1.35.E-04 | 0.39 |
| ZMYM3 | 5.32.E-04 | 1.34.E-04 | 0.25 | HCFC1 | 8.68.E-05 | 3.37.E-05 | 0.39 |
| MATR3 | 1.89.E-03 | 4.80.E-04 | 0.25 | MCM2 | 5.40.E-04 | 2.10.E-04 | 0.39 |
| GTF3C5 | 1.27.E-04 | 3.24.E-05 | 0.26 | SUMO2 | 3.90.E-03 | 1.52.E-03 | 0.39 |
| PCNA | 1.59.E-03 | 4.12.E-04 | 0.26 | HNRNPM | 5.02.E-03 | 1.96.E-03 | 0.39 |
| TRIM28 | 1.16.E-02 | 3.03.E-03 | 0.26 | HNRNPAB | 1.91.E-03 | 7.46.E-04 | 0.39 |
| RPL19 | 6.59.E-04 | 1.76.E-04 | 0.27 | CDCA7L | 7.63.E-04 | 2.98.E-04 | 0.39 |
| THRAP3 | 1.19.E-04 | 3.23.E-05 | 0.27 | EIF1AX | 2.35.E-04 | 9.28.E-05 | 0.39 |
| DNAJC8 | 5.82.E-04 | 1.59.E-04 | 0.27 | QARS | 1.70.E-04 | 6.82.E-05 | 0.40 |
| CFDP1 | 2.39.E-04 | 6.54.E-05 | 0.27 | SYNCRIP | 3.69.E-04 | 1.49.E-04 | 0.40 |
| MCM5 | 1.16.E-03 | 3.26.E-04 | 0.28 | RBM39 | 4.72.E-04 | 1.91.E-04 | 0.40 |
| LUC7L3 | 2.87.E-04 | 8.09.E-05 | 0.28 | SSRP1 | 4.73.E-03 | 1.92.E-03 | 0.41 |
| TKT | 2.11.E-04 | 5.94.E-05 | 0.28 | CCDC124 | 2.03.E-04 | 8.25.E-05 | 0.41 |
| KIF4A | 6.45.E-04 | 1.84.E-04 | 0.28 | MCM6 | 1.04.E-03 | 4.23.E-04 | 0.41 |
| TES | 1.77.E-04 | 5.17.E-05 | 0.29 | SPTBN1 | 1.04.E-03 | 4.24.E-04 | 0.41 |
| MSN | 2.48.E-04 | 7.38.E-05 | 0.30 | PPM1G | 1.14.E-04 | 4.63.E-05 | 0.41 |
| SRRM2 | 1.94.E-03 | 5.81.E-04 | 0.30 | Ku80 | 3.79.E-03 | 1.56.E-03 | 0.41 |

**Supplementary Table S1 (continued).** Proteins decreased on nascent DNA in *CDCA7* KO HEK293 cells determined by iPOND–MS/MS analysis (≤0.66). Related to Figure 1a.

| Protein | *WT (norm MS/MS score) | **CDCA7* KO (norm MS/MS score) | KO/WT |
|---|---|---|---|
| SAFB | 6.18.E-04 | 2.55.E-04 | 0.41 |
| RALY | 1.24.E-03 | 5.14.E-04 | 0.41 |
| BAZ1B | 2.60.E-03 | 1.08.E-03 | 0.42 |
| MSH2 | 2.61.E-03 | 1.10.E-03 | 0.42 |
| RPL7 | 2.76.E-04 | 1.16.E-04 | 0.42 |
| SEPTIN2 | 5.15.E-04 | 2.17.E-04 | 0.42 |
| CCT6A | 1.48.E-04 | 6.27.E-05 | 0.42 |
| ILF3 | 4.77.E-03 | 2.03.E-03 | 0.42 |
| SNRNP70 | 6.34.E-04 | 2.70.E-04 | 0.43 |
| FHL1 | 8.89.E-04 | 3.79.E-04 | 0.43 |
| STAG2 | 1.06.E-03 | 4.56.E-04 | 0.43 |
| CHD4 | 3.03.E-03 | 1.31.E-03 | 0.43 |
| PTBP1 | 1.62.E-03 | 7.06.E-04 | 0.44 |
| ATRX | 9.13.E-05 | 3.99.E-05 | 0.44 |
| BUB3 | 1.96.E-04 | 8.60.E-05 | 0.44 |
| SRSF6 | 3.93.E-04 | 1.72.E-04 | 0.44 |
| ZC3H18 | 2.01.E-04 | 8.83.E-05 | 0.44 |
| TOP2A | 3.38.E-03 | 1.48.E-03 | 0.44 |
| AIMP1 | 4.34.E-04 | 1.90.E-04 | 0.44 |
| SUPT16H | 3.94.E-03 | 1.73.E-03 | 0.44 |
| EHMT2 | 3.58.E-04 | 1.59.E-04 | 0.44 |
| H2AFY2 | 8.28.E-04 | 3.69.E-04 | 0.45 |
| TRIR | 4.76.E-04 | 2.12.E-04 | 0.45 |
| MDC1 | 1.01.E-03 | 4.49.E-04 | 0.45 |
| MCM7 | 1.79.E-03 | 8.08.E-04 | 0.45 |
| PSMA1 | 9.63.E-05 | 4.36.E-05 | 0.45 |
| SRSF3 | 5.08.E-03 | 2.32.E-03 | 0.46 |
| DHX15 | 3.36.E-04 | 1.53.E-04 | 0.46 |
| YBX1 | 2.76.E-04 | 1.27.E-04 | 0.46 |
| CCAR2 | 1.65.E-03 | 7.59.E-04 | 0.46 |
| PRKDC | 4.65.E-03 | 2.15.E-03 | 0.46 |
| KHDRBS1 | 2.59.E-03 | 1.20.E-03 | 0.46 |
| RPS9 | 5.04.E-04 | 2.33.E-04 | 0.46 |
| Ku70 | 2.05.E-03 | 9.49.E-04 | 0.46 |
| SF3B2 | 1.35.E-04 | 6.26.E-05 | 0.46 |
| HDGFL2 | 9.20.E-04 | 4.28.E-04 | 0.47 |
| PPIA | 3.87.E-03 | 1.82.E-03 | 0.47 |
| HNRNPA0 | 2.59.E-04 | 1.23.E-04 | 0.47 |
| BAZ1A | 1.52.E-03 | 7.26.E-04 | 0.48 |
| KIF22 | 1.59.E-04 | 7.58.E-05 | 0.48 |
| RPL4 | 1.11.E-03 | 5.31.E-04 | 0.48 |
| SRSF7 | 3.19.E-03 | 1.53.E-03 | 0.48 |
| LIG3 | 4.74.E-04 | 2.27.E-04 | 0.48 |
| DDX17 | 2.88.E-03 | 1.38.E-03 | 0.48 |
| HSP90AA1 | 1.23.E-03 | 5.92.E-04 | 0.48 |
| PPP1CC | 3.18.E-04 | 1.53.E-04 | 0.48 |
| Histone H4 | 1.61.E-01 | 7.78.E-02 | 0.48 |
| RBM4 | 5.44.E-04 | 2.63.E-04 | 0.48 |
| CCT2 | 9.23.E-04 | 4.47.E-04 | 0.48 |
| CAND1 | 8.11.E-04 | 3.93.E-04 | 0.49 |
| SNRNP200 | 9.27.E-04 | 4.51.E-04 | 0.49 |
| MSH6 | 1.93.E-03 | 9.40.E-04 | 0.49 |
| TOP2B | 6.70.E-04 | 3.28.E-04 | 0.49 |
| MCM3 | 2.53.E-03 | 1.25.E-03 | 0.49 |
| HNRNPD | 4.48.E-03 | 2.21.E-03 | 0.49 |
| Histone H2B | 4.49.E-02 | 2.22.E-02 | 0.49 |
| SNRPA1 | 8.99.E-04 | 4.46.E-04 | 0.50 |
| DDX5 | 3.32.E-03 | 1.65.E-03 | 0.50 |
| HMGB3 | 4.10.E-03 | 2.05.E-03 | 0.50 |
| SMARCA5 | 1.50.E-03 | 7.46.E-04 | 0.50 |
| PSIP1 | 3.62.E-03 | 1.81.E-03 | 0.50 |
| DDX39B | 2.09.E-03 | 1.05.E-03 | 0.50 |
| RPA1 | 4.76.E-04 | 2.39.E-04 | 0.50 |
| CHAF1A | 4.78.E-04 | 2.42.E-04 | 0.51 |
| HNRNPC | 8.27.E-03 | 4.19.E-03 | 0.51 |
| ELAVL1 | 2.54.E-03 | 1.29.E-03 | 0.51 |
| HNRNPUL2 | 4.01.E-04 | 2.05.E-04 | 0.51 |
| RPL29 | 4.78.E-04 | 2.44.E-04 | 0.51 |
| RBBP7 | 7.45.E-04 | 3.82.E-04 | 0.51 |
| SMC3 | 2.10.E-03 | 1.08.E-03 | 0.51 |
| TLN1 | 4.70.E-04 | 2.43.E-04 | 0.52 |
| CDCA5 | 9.68.E-05 | 5.00.E-05 | 0.52 |
| DNMT1 | 4.89.E-03 | 2.53.E-03 | 0.52 |
| RPS18 | 2.37.E-03 | 1.23.E-03 | 0.52 |
| RPS19 | 2.64.E-03 | 1.37.E-03 | 0.52 |
| HNRNPK | 1.34.E-02 | 6.98.E-03 | 0.52 |
| RBBP4 | 1.57.E-03 | 8.21.E-04 | 0.52 |
| PRDX3 | 1.93.E-04 | 1.01.E-04 | 0.52 |
| TLE4 | 2.03.E-04 | 1.06.E-04 | 0.53 |
| SRSF1 | 2.97.E-03 | 1.56.E-03 | 0.53 |
| DNAJA2 | 2.21.E-04 | 1.16.E-04 | 0.53 |
| RING1 | 2.58.E-04 | 1.37.E-04 | 0.53 |
| YTHDF2 | 6.65.E-05 | 3.52.E-05 | 0.53 |
| U2SURP | 2.04.E-04 | 1.08.E-04 | 0.53 |
| RPS14 | 4.72.E-04 | 2.51.E-04 | 0.53 |
| RCOR1 | 4.78.E-05 | 2.54.E-05 | 0.53 |
| HNRNPDL | 3.96.E-03 | 2.11.E-03 | 0.53 |
| CELF1 | 2.42.E-04 | 1.29.E-04 | 0.53 |
| MCM4 | 1.34.E-03 | 7.15.E-04 | 0.53 |
| HNRNPU | 7.28.E-03 | 3.90.E-03 | 0.54 |
| ACP1 | 7.23.E-04 | 3.88.E-04 | 0.54 |
| RPL7A | 5.55.E-04 | 2.98.E-04 | 0.54 |
| MRE11 | 2.44.E-04 | 1.31.E-04 | 0.54 |
| PFN1 | 1.63.E-03 | 8.79.E-04 | 0.54 |
| U2AF2 | 1.36.E-03 | 7.32.E-04 | 0.54 |
| RARS | 9.31.E-05 | 5.01.E-05 | 0.54 |
| KHSRP | 2.97.E-03 | 1.61.E-03 | 0.54 |
| CHAF1B | 1.60.E-03 | 8.66.E-04 | 0.54 |
| SRSF2 | 2.35.E-03 | 1.27.E-03 | 0.54 |
| KPNA2 | 2.43.E-04 | 1.31.E-04 | 0.54 |
| EFTUD2 | 6.90.E-04 | 3.76.E-04 | 0.54 |
| SNU13 | 1.19.E-04 | 6.52.E-05 | 0.55 |
| NAP1L1 | 4.16.E-04 | 2.28.E-04 | 0.55 |
| SNRPD3 | 8.01.E-04 | 4.39.E-04 | 0.55 |
| TFAM | 3.98.E-04 | 2.19.E-04 | 0.55 |
| RPL38 | 9.45.E-04 | 5.19.E-04 | 0.55 |
| DDX1 | 4.48.E-04 | 2.47.E-04 | 0.55 |
| PCBP1 | 1.20.E-03 | 6.66.E-04 | 0.56 |
| SNRPD1 | 6.20.E-04 | 3.44.E-04 | 0.56 |
| C1QBP | 3.45.E-04 | 1.92.E-04 | 0.56 |
| ERH | 1.79.E-03 | 9.99.E-04 | 0.56 |
| RFC4 | 1.41.E-03 | 8.01.E-04 | 0.57 |
| RPL22 | 3.15.E-04 | 1.79.E-04 | 0.57 |
| HNRNPH1 | 1.67.E-03 | 9.53.E-04 | 0.57 |
| H2AFV | 4.04.E-03 | 2.30.E-03 | 0.57 |
| RBMX | 3.60.E-03 | 2.06.E-03 | 0.57 |
| CCAR1 | 4.51.E-04 | 2.59.E-04 | 0.57 |
| PDS5B | 4.59.E-04 | 2.64.E-04 | 0.58 |
| RPS13 | 5.19.E-04 | 2.99.E-04 | 0.58 |
| TNPO1 | 7.19.E-04 | 4.15.E-04 | 0.58 |

**Supplementary Table S1 (continued).** Proteins decreased on nascent DNA in *CDCA7* KO HEK293 cells determined by iPOND–MS/MS analysis (≤0.66). Related to Figure 1a.

| Protein | *WT (norm MS/MS score) | **CDCA7* KO (norm MS/MS score) | KO/WT |
|---|---|---|---|
| RCC2 | 2.01.E-03 | 1.16.E-03 | 0.58 |
| PPA1 | 1.41.E-04 | 8.20.E-05 | 0.58 |
| HMGB2 | 9.64.E-04 | 5.64.E-04 | 0.59 |
| SUB1 | 5.82.E-04 | 3.43.E-04 | 0.59 |
| PARP1 | 1.38.E-02 | 8.14.E-03 | 0.59 |
| HNRNPA2B1 | 8.67.E-03 | 5.13.E-03 | 0.59 |
| YWHAE | 1.21.E-03 | 7.17.E-04 | 0.59 |
| PRDX5 | 2.41.E-04 | 1.43.E-04 | 0.60 |
| ATAD2 | 7.24.E-04 | 4.31.E-04 | 0.60 |
| UHRF1 | 9.47.E-04 | 5.69.E-04 | 0.60 |
| DIS3 | 1.53.E-04 | 9.27.E-05 | 0.61 |
| RPS4X | 2.25.E-03 | 1.37.E-03 | 0.61 |
| RUVBL1 | 1.74.E-03 | 1.06.E-03 | 0.61 |
| FUS | 1.76.E-03 | 1.07.E-03 | 0.61 |
| POGZ | 1.52.E-04 | 9.25.E-05 | 0.61 |
| SMC1A | 1.98.E-03 | 1.21.E-03 | 0.61 |
| RPS25 | 2.34.E-03 | 1.43.E-03 | 0.61 |
| HNRNPL | 1.81.E-03 | 1.11.E-03 | 0.61 |
| FEN1 | 2.17.E-03 | 1.34.E-03 | 0.62 |
| ACTN1 | 1.53.E-04 | 9.42.E-05 | 0.62 |
| CALM3 | 5.75.E-04 | 3.57.E-04 | 0.62 |
| RPL24 | 4.60.E-04 | 2.86.E-04 | 0.62 |
| NME2 | 4.08.E-04 | 2.54.E-04 | 0.62 |
| TPM3 | 1.16.E-03 | 7.23.E-04 | 0.62 |
| HNRNPF | 8.76.E-04 | 5.46.E-04 | 0.62 |
| CTBP1 | 1.50.E-04 | 9.43.E-05 | 0.63 |
| PHGDH | 1.10.E-03 | 6.91.E-04 | 0.63 |
| HP1BP3 | 1.96.E-03 | 1.24.E-03 | 0.63 |
| NUMA1 | 5.83.E-05 | 3.70.E-05 | 0.64 |
| GLRX3 | 1.65.E-04 | 1.05.E-04 | 0.64 |
| CDC5L | 1.98.E-04 | 1.26.E-04 | 0.64 |
| GCN1 | 1.33.E-04 | 8.45.E-05 | 0.64 |
| RPS6 | 6.31.E-04 | 4.02.E-04 | 0.64 |
| SUZ12 | 1.53.E-04 | 9.74.E-05 | 0.64 |
| LDHB | 1.72.E-04 | 1.10.E-04 | 0.64 |
| SNRPB2 | 1.56.E-04 | 1.00.E-04 | 0.64 |
| RPL17 | 1.41.E-03 | 9.03.E-04 | 0.64 |
| RPL23A | 1.41.E-03 | 9.09.E-04 | 0.64 |
| PHF14 | 8.35.E-05 | 5.37.E-05 | 0.64 |
| RUVBL2 | 1.80.E-03 | 1.17.E-03 | 0.65 |
| PGK1 | 7.86.E-04 | 5.11.E-04 | 0.65 |
| ANP32B | 6.88.E-04 | 4.48.E-04 | 0.65 |
| RTCB | 8.72.E-04 | 5.68.E-04 | 0.65 |
| CSE1L | 1.11.E-03 | 7.21.E-04 | 0.65 |
| PRMT1 | 3.98.E-04 | 2.60.E-04 | 0.65 |
| ACTG1 | 6.62.E-02 | 4.32.E-02 | 0.65 |
| IQGAP1 | 1.29.E-04 | 8.45.E-05 | 0.65 |
| RPL8 | 9.24.E-04 | 6.06.E-04 | 0.66 |
| RPS2 | 5.09.E-04 | 3.34.E-04 | 0.66 |
| PCBP2 | 2.66.E-03 | 1.75.E-03 | 0.66 |
| IPO5 | 1.55.E-04 | 1.02.E-04 | 0.66 |
| PHB2 | 7.69.E-04 | 5.08.E-04 | 0.66 |
| RAD50 | 8.74.E-05 | 5.79.E-05 | 0.66 |
| PSMC2 | 7.45.E-05 | 4.94.E-05 | 0.66 |
| RPS23 | 1.33.E-04 | 8.84.E-05 | 0.66 |
| H2AFY | 4.18.E-03 | 2.78.E-03 | 0.66 |

*Normalized MS/MS scores of each protein by total MS/MS scores of all proteins.

**Supplementary Table S2.** Proteins increased on nascent DNA in *CDCA7* KO HEK293 cells determined by iPOND–MS/MS analysis (≥1.5). Related to Figure 1a.

| Protein | Description | *WT (norm MS/MS score) | **CDCA7* KO (norm MS/MS score) | KO/WT |
|---|---|---|---|---|
| ZC3H11A | Zinc finger CCCH domain-containing protein 11A | 3.10.E-05 | 6.95.E-04 | 22.38 |
| PDHA1 | Pyruvate dehydrogenase E1 component subunit alpha, somatic form, mitochondrial | 3.48.E-06 | 5.40.E-05 | 15.50 |
| GTF3C3 | General transcription factor 3C polypeptide 3 | 5.75.E-06 | 7.17.E-05 | 12.47 |
| DCD | Dermcidin | 1.31.E-03 | 7.83.E-03 | 5.99 |
| HRNR | Hornerin | 1.87.E-04 | 8.30.E-04 | 4.44 |
| DLAT | Dihydrolipoyllysine-residue acetyltransferase component of pyruvate dehydrogenase complex, mitochondrial | 4.72.E-05 | 1.82.E-04 | 3.85 |
| LMNB1 | Lamin-B1 | 3.84.E-04 | 1.25.E-03 | 3.26 |
| ACACA | Acetyl-CoA carboxylase 1 | 2.17.E-03 | 6.46.E-03 | 2.98 |
| LMNA | Prelamin-A/C | 1.96.E-04 | 5.75.E-04 | 2.93 |
| VDAC2 | Voltage-dependent anion-selective channel protein 2 | 6.78.E-04 | 1.80.E-03 | 2.66 |
| MDH2 | Malate dehydrogenase, mitochondrial | 3.99.E-04 | 9.97.E-04 | 2.50 |
| SNW1 | SNW domain-containing protein 1 | 1.58.E-04 | 3.93.E-04 | 2.49 |
| DSP | Desmoplakin | 4.79.E-04 | 1.15.E-03 | 2.40 |
| PC | Pyruvate carboxylase, mitochondrial | 9.43.E-04 | 2.15.E-03 | 2.28 |
| PCCA | Propionyl-CoA carboxylase alpha chain, mitochondrial | 1.09.E-03 | 2.35.E-03 | 2.16 |
| MARS | Methionine--tRNA ligase, cytoplasmic | 1.64.E-04 | 3.55.E-04 | 2.16 |
| PRRC2C | Protein PRRC2C | 1.35.E-04 | 2.81.E-04 | 2.07 |
| LMNB2 | Lamin-B2 | 9.58.E-05 | 1.96.E-04 | 2.05 |
| NPM1 | Nucleophosmin | 2.51.E-04 | 5.04.E-04 | 2.01 |
| VDAC3 | Voltage-dependent anion-selective channel protein 3 | 3.80.E-05 | 6.70.E-05 | 1.77 |
| RPLP0P6 | 60S acidic ribosomal protein P0-like | 6.25.E-04 | 1.08.E-03 | 1.73 |
| PUM1 | Pumilio homolog 1 | 4.98.E-05 | 8.55.E-05 | 1.72 |
| TMPO | Lamina-associated polypeptide 2, isoforms beta/gamma | 3.54.E-04 | 5.84.E-04 | 1.65 |
| ACLY | ATP-citrate synthase | 1.01.E-04 | 1.65.E-04 | 1.63 |
| PCCB | Propionyl-CoA carboxylase beta chain, mitochondrial | 6.09.E-04 | 9.68.E-04 | 1.59 |
| HACD3 | Very-long-chain (3R)-3-hydroxyacyl-CoA dehydratase 3 | 4.47.E-04 | 6.99.E-04 | 1.57 |
| LRPPRC | Leucine-rich PPR motif-containing protein, mitochondrial | 1.14.E-03 | 1.71.E-03 | 1.50 |

*Normalized MS/MS scores of each protein by total MS/MS scores of all proteins.

**Supplementary Table S3.** KEGG pathway analysis of the 296 proteins decreased on nascent DNA in *CDCA7* KO HEK293 cells. Related to Figure 1c.

| **Term** | ***P* value** | **Proteins** |
|---|---|---|
| Spliceosome | 2.0.E-37 | CDC5L, DDX5, DHX15, DDX23, DDX39B, EFTUD2, EIF4A3, HNRPA3, HNRPC, HNRNPK, HNRPM, HNRPU, PCBP1, PRPF3, PRPF6, PRPF8, RBMX, SRSF1, SRSF2, SRSF3, SRSF5, SRSF6, SRSF7, SNRPD1, SNRPD3, SNRNP70, SNRNP200, SNRPA1, SNRPB2, SNRPC, SF3A1, SF3B1, SF3B2, SF3B3, SNU13, TCERG1, THOC2, THOC4, U2AF2, U2SURO |
| DNA replication | 3.1.E-11 | FEN1, MCM2, MCM3, MCM4, MCM5, MCM6, MCM7, PCNA, POLA1, RFC2, RFC4, RPA |
| Cell cycle | 6.1.E-08 | BUB3, HDAC2, MCM2, MCM3, MCM4, MCM5, MCM6, MCM7, PCNA, PRKDC, RAD21, STAG2, SMC1A, SMC3, YWHAE |
| Non-homologous end joining | 2.2.E-06 | FEN1, Ku70, Ku80, MRE11, PRKDC, RAD50 |
| RNA transport | 2.0.E-04 | DDX39B, E1F1AX, EIF3B, EIF4A3, PNN, PABPC3, RNPS1, SRRM1, SUMO1, SUMO2, THOC2, THOC4 |
| Herpes simplex infection | 2.3.E-04 | C1QBP, CSNK2B, GTF2I, HNRNPK, HCFC1, PPP1CC, SRSF1, SRSF2, SRSF3, SRSF5, SRSF6, SRSF7, THOC4 |
| Mismatch repair | 6.2.E-04 | MSH6, PCNA, RFC2, RFC3, RPA1 |
| mRNA surveillance pathway | 1.6.E-03 | DDX39B, RNPS1, EIF4A3, PNN, PABPC3, PPP1CC, SRRM1, THOC4 |
| Base excision repair | 4.1.E-03 | FEN1, LIG3, PARP1, PCNA, XRCC1 |
| Nucleotide excision repair | 8.5.E-03 | DDB1, PCNA, RFC2, RFC4, RPA1 |

**Supplementary Table S4** Proteins coimmunoprecipitated with CDCA7 (peptide ≥ 1.0) and were decreased on nascent DNA in *CDCA7* KO HEK293 cells (≤0.66). Related to Fig. 1d,e.

| Protein | Description | MW | *WT (iPOND) | **CDCA7* KO (iPOND) | KO/WT (iPOND) | **CDCA7_WT (co-IPed) | **CDCA7_R274C (co-IPed) |
|---|---|---|---|---|---|---|---|
| Histone H2B | Histone H2B | 13,898 | 4.49.E-02 | 2.22.E-02 | 0.49 | 28 | 7 |
| DDX21 | Nucleolar RNA helicase 2 | 87,804 | 1.15.E-04 | 2.26.E-05 | 0.20 | 16 | 14 |
| Histone H4 | Histone H4 | 11,360 | 1.61.E-01 | 7.78.E-02 | 0.48 | 16 | 7 |
| Ku80 | X-ray repair cross-complementing protein 5 | 83,222 | 3.79.E-03 | 1.56.E-03 | 0.41 | 10 | 1 |
| SMARCA5 | SWI/SNF-related matrix-associated actin-dependent regulator of chromatin subfamily A member 5 | 122,513 | 1.50.E-03 | 7.46.E-04 | 0.50 | 9 | 5 |
| SUPT16H | FACT complex subunit SPT16 | 120,409 | 3.94.E-03 | 1.73.E-03 | 0.44 | 7 | 2 |
| PRKDC | DNA-dependent protein kinase catalytic subunit | 473,749 | 4.65.E-03 | 2.15.E-03 | 0.46 | 5 | 1 |
| RFC2 | Replication factor C subunit 2 | 39,588 | 2.55.E-04 | 7.73.E-05 | 0.30 | 4 | 2 |
| RTCB | tRNA-splicing ligase RtcB homolog | 55,688 | 8.72.E-04 | 5.68.E-04 | 0.65 | 4 | 1 |
| RBBP7 | Histone-binding protein RBBP7 | 48,132 | 7.45.E-04 | 3.82.E-04 | 0.51 | 4 | 0 |
| RBM4 | RNA-binding protein 4 | 40,688 | 5.44.E-04 | 2.63.E-04 | 0.48 | 3 | 0 |
| RBBP4 | Histone-binding protein RBBP4 | 47,911 | 1.57.E-03 | 8.21.E-04 | 0.52 | 2 | 3 |
| RALY | RNA-binding protein Raly | 32,501 | 1.24.E-03 | 5.14.E-04 | 0.41 | 2 | 1 |
| RFC4 | Replication factor C subunit 4 | 40,170 | 1.41.E-03 | 8.01.E-04 | 0.57 | 2 | 1 |
| SSRP1 | FACT complex subunit SSRP1 | 81,367 | 4.73.E-03 | 1.92.E-03 | 0.41 | 2 | 1 |
| THOC4 | THO complex subunit 4 | 26,872 | 1.72.E-04 | 6.29.E-06 | 0.04 | 2 | 1 |
| BUB3 | Mitotic checkpoint protein BUB3 | 37,587 | 1.96.E-04 | 8.60.E-05 | 0.44 | 2 | 0 |
| TFAM | Transcription factor A, mitochondrial | 29,306 | 3.98.E-04 | 2.19.E-04 | 0.55 | 2 | 0 |
| TPX2 | Targeting protein for Xklp2 | 86,227 | 5.04.E-04 | 1.71.E-05 | 0.03 | 2 | 0 |
| PHB2 | Prohibitin-2 | 33,276 | 7.69.E-04 | 5.08.E-04 | 0.66 | 1 | 3 |
| SRSF1 | Serine/arginine-rich splicing factor 1 | 27,842 | 2.97.E-03 | 1.56.E-03 | 0.53 | 1 | 3 |
| MRE11 | Double-strand break repair protein MRE11 | 80,885 | 2.44.E-04 | 1.31.E-04 | 0.54 | 1 | 2 |
| PRPF3 | U4/U6 small nuclear ribonucleoprotein Prp3 | 77,652 | 1.03.E-04 | 1.69.E-05 | 0.16 | 1 | 2 |
| RCC2 | Protein RCC2 | 56,790 | 2.01.E-03 | 1.16.E-03 | 0.58 | 1 | 2 |
| C1QBP | Complement component 1 Q subcomponent-binding protein, mitochondrial | 31,742 | 3.45.E-04 | 1.92.E-04 | 0.56 | 1 | 1 |
| CBX3 | Chromobox protein homolog 3 | 20,969 | 4.56.E-03 | 1.62.E-03 | 0.36 | 1 | 1 |
| MTA1 | Metastasis-associated protein MTA1 | 81,420 | 3.60.E-04 | 1.27.E-04 | 0.35 | 1 | 1 |
| SUZ12 | Polycomb protein SUZ12 | 83,744 | 1.53.E-04 | 9.74.E-05 | 0.64 | 1 | 1 |
| ADNP | Activity-dependent neuroprotector homeobox protein | 124,854 | 1.05.E-03 | 1.33.E-04 | 0.13 | 1 | 0 |
| BAZ1A | Bromodomain adjacent to zinc finger domain protein 1A | 180,246 | 1.52.E-03 | 7.26.E-04 | 0.48 | 1 | 0 |
| DEK | Protein DEK | 42,933 | 4.93.E-03 | 1.80.E-03 | 0.36 | 1 | 0 |
| H2AFV | Histone H2A.V (H2AZ2) | 13,501 | 4.04.E-03 | 2.30.E-03 | 0.57 | 1 | 0 |
| H2AFY | Histone macro-H2A.1 | 39,764 | 4.18.E-03 | 2.78.E-03 | 0.66 | 1 | 0 |
| Ku70 | X-ray repair cross-complementing protein 6 | 70,084 | 2.05.E-03 | 9.49.E-04 | 0.46 | 1 | 0 |
| MCM4 | DNA replication licensing factor MCM4 | 97,068 | 1.34.E-03 | 7.15.E-04 | 0.53 | 1 | 0 |
| MCM7 | DNA replication licensing factor MCM7 | 81,884 | 1.79.E-03 | 8.08.E-04 | 0.45 | 1 | 0 |
| MDC1 | Mediator of DNA damage checkpoint protein 1 | 227,669 | 1.01.E-03 | 4.49.E-04 | 0.45 | 1 | 0 |
| PGK1 | Phosphoglycerate kinase 1 | 44,985 | 7.86.E-04 | 5.11.E-04 | 0.65 | 1 | 0 |
| RAD50 | DNA repair protein RAD50 | 154,823 | 8.74.E-05 | 5.79.E-05 | 0.66 | 1 | 0 |
| SRSF3 | Serine/arginine-rich splicing factor 3 | 19,546 | 5.08.E-03 | 2.32.E-03 | 0.46 | 1 | 0 |
| TOP2A | DNA topoisomerase 2-alpha | 175,017 | 3.38.E-03 | 1.48.E-03 | 0.44 | 1 | 0 |
| UHRF1 | Ubiquitin like with PHD and RING finger domains 1 | 91,297 | 9.47.E-04 | 5.69.E-04 | 0.60 | 1 | 0 |

*Normalized MS/MS scores of each protein by total MS/MS scores of all proteins.

**Peptide numbers coimmunoprecipitated with FLAG-CDCA7 WT and/or R274C[13].

**Supplementary Table S5.** Antibodies used in the present study.

| Name | Company | Catalog# | Clone# | Dilution |
|---|---|---|---|---|
| Anti-PCNA mouse monoclonal antibody | BioLegend | 307902 | PC-10 | 1:1000 |
| Anti-DNA:RNA mouse monoclonal antibody | Millipore | MABE1095 | S9.6 | 1:200 |
| Anti-V5 mouse monoclonal antibody | Wako | 011-23591 | 6F5 | 1:1000 |
| Anti-V5 rabbit polyclonal antibody | Abcam | ab9116 | - | 1:1000 |
| Anti-γH2AX rabbit monoclonal antibody | Abcam | ab81299 | EP854(2)Y | 1:1000 |
| anti-DDX21 rabbit polyclonal antibody | Proteintech | 10528-1-AP | - | 1:1000 |
| anti-53BP1 rabbit polyclonal antibody | Cell Signaling Technology | 4937 | - | 1:1000 |
| anti-SUPT16H rabbit monoclonal antibody | Abcam | Ab108960 | EPR3685 | 1:1000 |
| Anti-UHRF1 mouse monoclonal antibody | BD Biosciences | 612264 | 28/ICBP90 | 1:1000 |
| Anti-Ku80 mouse monoclonal antibody | Abcam | ab119935 | 5C5 | 1:1000 |
| Anti-Histone H2B mouse monoclonal antibody | Abcam | ab52484 | mAbcam 52484 | 1:1000 |
| anti-RBBP7 rabbit monoclonal antibody | LifeSpan Biosciences | LS-C138282 | - | 1:1000 |
| anti-RBBP4 rabbit monoclonal antibody | LifeSpan Biosciences | LS-C105517 | - | 1:1000 |
| anti-H2AFY rabbit monoclonal antibody | Abcam | ab183041 | EPR9359(2) | 1:1000 |
| anti-Rad50 rabbit polyclonal antibody | Cell Signaling Technology | 3427 | - | 1:1000 |
| anti-MCM2 rabbit monoclonal antibody | Cell Signaling Technology | 3619 | D7G11 | 1:1000 |
| anti-CDCA7 rabbit polyclonal antibody | MBL | MB-148 | - | 1:1000 |
| anti-ACTB mouse monoclonal antibody | Santa Cruz Biotechnology | sc-69879 | AC-15 | 1:1000 |

**Supplementary Table S6.** Primers for RT-qPCR, DRIP qPCR, R-ChIP qPCR, and ChIP qPCR.

| Gene or genomic region | Forward primer | Reverse primer | Experiments |
|---|---|---|---|
| $\alpha$-satellite | 5′-GCTTTAAGGTCAATGGCAGAA-3′ | 5′-ACCATTGACCTCAAAGCGGC-3′ | RT-qPCR, DRIP qPCR, R-ChIP-qPCR, ChIP qPCR |
| Satellite-2 | 5′-AATGAAAGGAGTCATTATCTAATG-3′ | 5′-ATTCCATTCCATTAGATGATTCC-3′ | RT-qPCR, DRIP qPCR, R-ChIP-qPCR, ChIP qPCR |
| *TFPT* | 5′-TCTGGGAGTCCAAGCAGACT-3′ | 5′-AAGGAGCCACTGAAGGGTTT-3′ | DRIP qPCR, R-ChIP qPCR, ChIP qPCR |
| *CALM3* | 5′-GAGGAATTGTGGCGTTGACT-3′ | 5′-AGAGTGGCCAAATGAGCAGT-3′ | DRIP qPCR, R-ChIP qPCR, ChIP qPCR |
| *EGR1 neg* | 5′-GAACGTTCAGCCTCGTTCTC-3′ | 5′-GGAAGGTGGAAGGAAACACA-3′ | DRIP qPCR, R-ChIP qPCR, ChIP qPCR |
| *SNRPN neg* | 5′-GCCAAATGAGTGAGGATGGT-3′ | 5′-TCCTCTCTGCCTGACTCCAT-3′ | DRIP qPCR, R-ChIP qPCR, ChIP qPCR |
| 18S rRNA | 5′-GTAACCCGTTGAACCCCATT-3′ | 5′-CCATCCAATCGGTAGTAGCG-3′ | RT-qPCR |
| $Amp^R$ | 5′-CCGGCTCCAGATTTATCAGC-3′ | 5′-ACTCTAGCTTCCCGGCAAC-3′ | R-ChIP qPCR |

**Unedited gel images**

**Unedited gel for Figure 1b.**

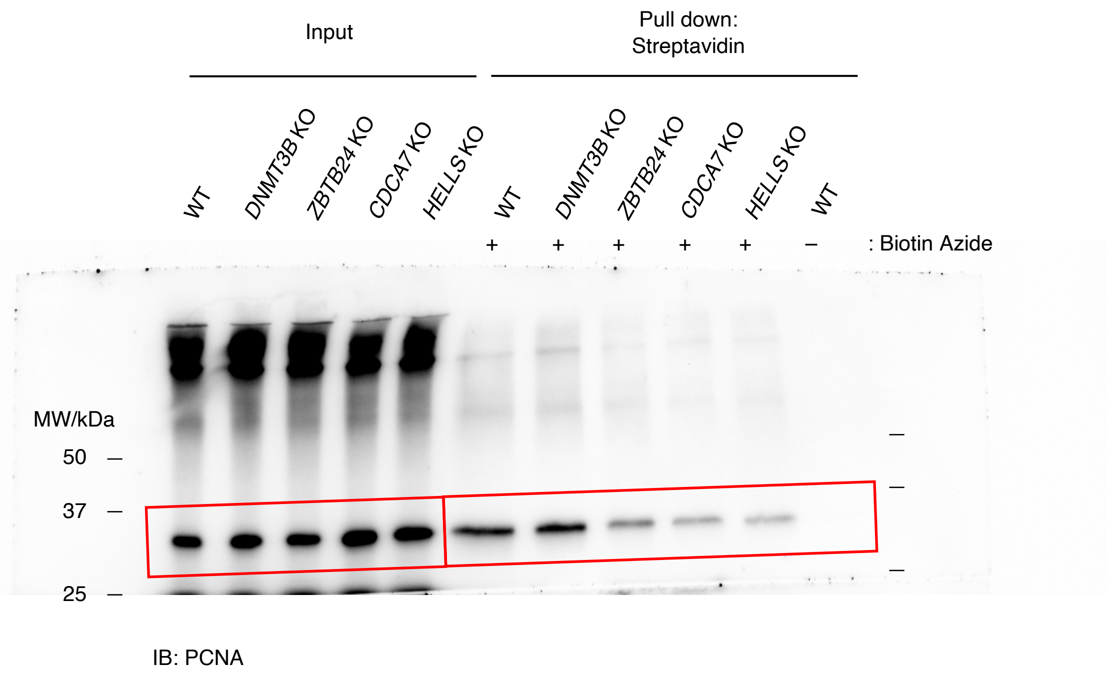

**Unedited image for Supplementary Figure S1.**

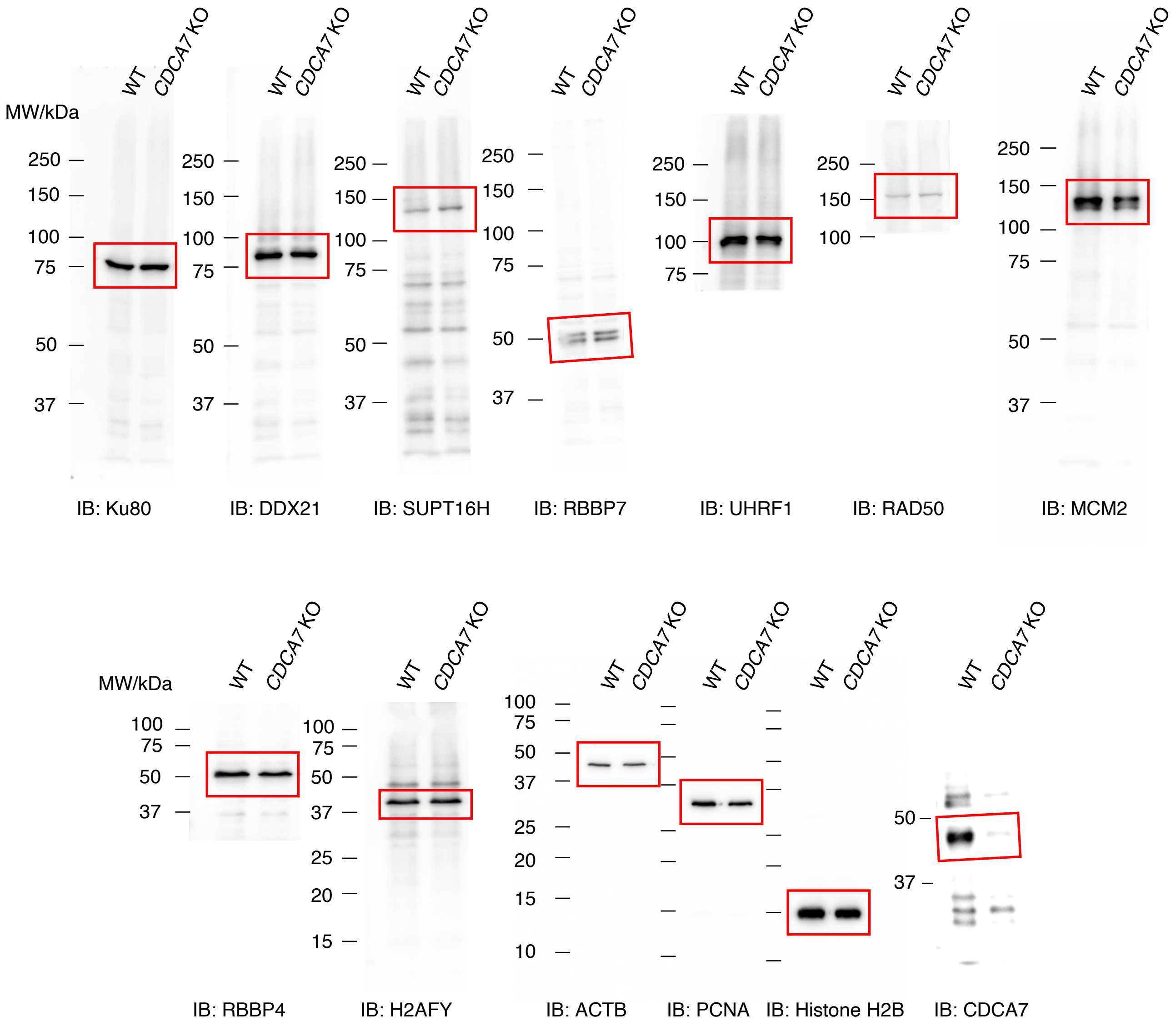

Supplement: Supplementary file 1 — Supplementary Information. (32324 kb) [file 41598_2020_74636_MOESM1_ESM.pdf]
